# Molecular profiling of skin cells identifies distinct cellular signatures in radiation-induced skin injury across various stages in the murine dataset

**DOI:** 10.1186/s40164-025-00596-w

**Published:** 2025-02-25

**Authors:** Hongxuan Yu, Tao Zhong, Ying Xu, Zengfu Zhang, Jiachun Ma, Jupeng Yuan, Minglei Wang, Meng Wu, Jinming Yu, Yuequn Ma, Dawei Chen

**Affiliations:** 1 Shandong University Cancer Center, Shandong University, Jinan, Shandong China; 2 Department of Radiation Oncology, Shandong Provincial Key Laboratory of Precision Oncology, Shandong Cancer Hospital and Institute, Shandong First Medical University, Shandong Academy of Medical Sciences, Jinan, Shandong China; 3 Department of Radiation Oncology, The First Hospital of China Medical University, 155 N, Nanjing Street, Shenyang, Liaoning China

**Keywords:** Radiotherapy, Atlas, Radiation-induced skin injury, Single-cell RNA sequencing, Cellular signatures

## Abstract

**Background:**

Radiation-induced skin injury (RISI) commonly manifests in cancer patients undergoing radiotherapy (RT). However, a universally accepted standard for treating radiation injury has not yet been established. Our objective was to provide a detailed molecular overview of skin pre- and post-radiation therapy, aiming to enhance our understanding of the subclusters and molecular mechanisms contributing to radiodermatitis.

**Methods:**

C57BL/6 mice were subjected to a single fraction (20 Gy) of RT targeting the right dorsal skin. We then employed integrated single-cell RNA sequencing (scRNA-seq) to analyze skin samples from mice at 7 and 30 days after radiation exposure, as well as from non-irradiated mice. The Seurat analysis pipeline, Cellchat, SCP, and ssGSEA were used to define the cell types and mechanisms involved in radiation-induced skin injury. Reverse transcription polymerase chain reaction (RT-PCR), multiplex immunofluorescent staining, and other datasets (GSE130183, GSE193564, and GSE193807) were used to validate our findings.

**Results:**

Thirty-two distinct cell clusters encompassing 71,412 cells were identified. We discovered that cycling keratinocytes (KCs), with the BMP signaling pathway enriched, could activate the Wnt pathway, as well as the SMAD pathways, driving the wound healing and fibrosis processes in RISI. Terminally differentiated secretory-papillary fibroblasts (Fibs) are capable of attracting immune cells, which contributes to the pathogenesis of RISI. Lymphatic endothelial cells (ECs) with pro-inflammatory properties play a critical role in the pathogenesis of RISI by facilitating leukocyte migration. Our analysis also highlighted enhanced ligand-receptor interactions, notably the interactions between chemokines like CXCL10, CCL2, and ACKR1, across subclusters of inflammatory KCs, Fibs, ECs, and immune cells, underscoring their pivotal role in leukocyte recruitment in RISI.

**Conclusions:**

Cycling KCs, secretory-papillary Fibs, and lymphatic ECs play critical roles in RISI progression. Targeting the interactions of these subclusters with immune cells might help improve the severity of RISI. Furthermore, our study provides a valuable resource for understanding the interactions among immune cells in the context of RISI.

**Graphical Abstract:**

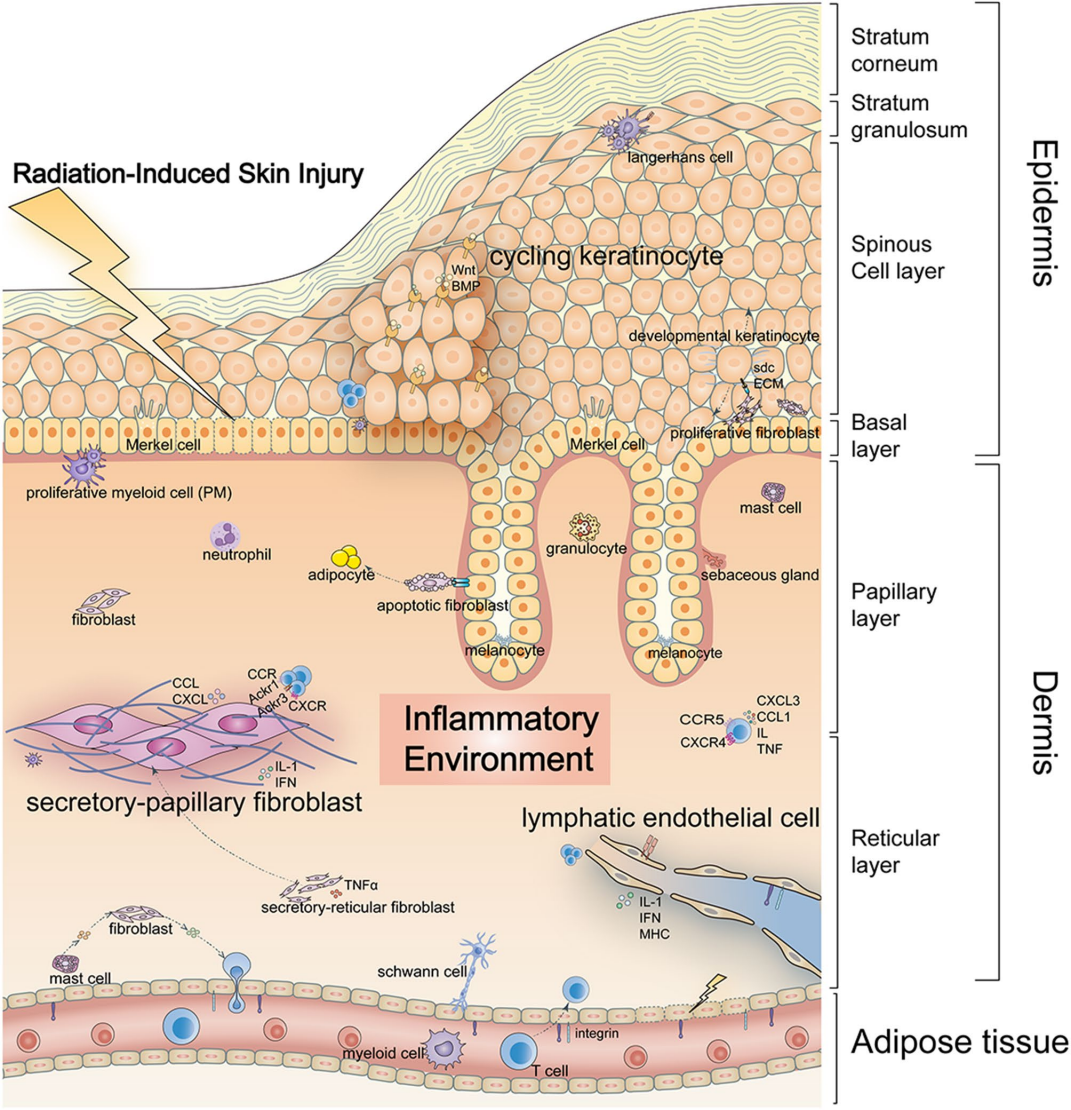

**Supplementary Information:**

The online version contains supplementary material available at 10.1186/s40164-025-00596-w.

## Highlights

A comprehensive molecular landscape in the skin pre- and post-radiation therapy is presented.

Cycling KCs, lymphatic ECs, and secretory-papillary Fibs are highly involved in RISI pathogenesis.

Immune cell sub-populations play substantial roles in inflammatory response in RISI.

**Supplementary Information:**

The online version contains supplementary material available at 10.1186/s40164-025-00596-w.

## Introduction

Radiation-induced skin injury (RISI) represents a notable complication in radiotherapy, predominantly affecting sensitive areas such as the face, neck, trunk, and extremities [1, 2]. The severity of RISI spans a spectrum, from mild erythema to severe manifestations such as moist desquamation and ulceration, influenced by factors like radiation type and dose [1, 2]. It is noteworthy that approximately 95% of individuals receiving radiotherapy for tumor treatment exhibit varying degrees of radiodermatitis [3–6]. Radiation-induced injuries can substantially affect patient’s quality of life and well-being, potentially resulting in therapy discontinuation and inadequate follow-up treatment. Despite advancements such as IMRT improving dose distribution [7], the limited knowledge of the inflammatory mechanisms of radiodermatitis and onset triggers hinders progress in mitigating these adverse side effects [1].

Skin reactions in radiodermatitis arise from a combination of direct DNA damage by reactive oxygen species (ROS) and ensuing inflammatory and immune response [8]. Recent studies highlight the involvement of diverse factors, cells, microbiota [9], and signaling pathways in the initiation of inflammatory, apoptotic, and oxidative responses during the development of radiodermatitis. These factors encompass the senescence-associated IL-6/CCR6 axis [10], the ROS-related Nrf2/GCH1/BH4 axis [11], and fatty acid metabolism [12]. Efforts to mitigate RISI have led to the development of diverse treatments, ranging from natural drugs to chemical-based medicines, targeting critical pathways and cytokines [13]. Nevertheless, the precise roles of skin cells, their disrupted intercommunication, and cellular mediators driving this inflammatory predisposition are yet to be fully characterized. Consequently, a universally accepted standard for treating radiation injury is still lacking.

The study aims to categorize radiation-induced skin injuries into acute and late (chronic) types. Acute injuries typically manifest within hours to weeks post radiation exposure, while late injuries develop over months to years to manifest [14]. To elucidate the distinct processes involved, skin samples were collected at two specific time points post-radiotherapy: day 7 and day 30. It is worth mentioning that ionizing radiation predominantly impacts the deeper dermis with relatively lesser effect on the upper epidermal layer [8]. Consequently, our research mainly focused on the clusters of fibroblasts and endotheliocytes located in deeper sites, alongside keratinocytes. Furthermore, we have also focused on the changes in both the quantity and function of rarer subclusters, including melanocytes, Merkel cells, mast cells, and Langerhans cells, in RISI. By analyzing cellular composition at a single-cell level in both normal and radiation-exposed skin samples, we aimed to thoroughly characterize the cellular composition and identify the key mediators responsible for the inflammatory changes associated with radiodermatitis progression.

## Methods

### Mouse skin samples

Our study included eighteen mouse samples. Initially, female C57BL/6 mice were subjected to a single fraction (20 Gy) of radiation therapy targeted at the right dorsal region, specifically above the right forelimb. Subsequent to the radiotherapy, mouse skin samples were collected at 7 and 30 days post-treatment for analysis using scRNA-seq and multiplexed immunofluorescence staining. The study was divided into three groups: Day 0, Day 7, and Day 30 post-radiotherapy. Notably, samples were obtained at three-time intervals, incorporating Day 0 skin tissues in all batches. Detailed information regarding the groups and treatment are provided in Table S1. The samples were acquired from the Department of Radiation Oncology, Shandong University Cancer Center, Jinan, Shandong, China, and the Department of Radiation Oncology and Shandong Provincial Key Laboratory of Precision Oncology, Shandong First Medical University and Shandong Academy of Medical Sciences, Jinan, Shandong, China.

### Single-cell suspension preparation

Approximately 300 mg skin tissue per group was processed using the Liver Dissociation Kit mouse (#130-105-807, Miltenyi Biotec Inc., Auburn, CA, USA). Tissue samples were dissected into small pieces (< 1 mm3) and transferred to the gentleMACSC Tube containing the enzyme mix. Subsequently, the tissues were processed using the gentleMACS Dissociator (Miltenyi Biotec Inc., Auburn, CA, USA). Following this, 2 mL DMEM was added for dilution and the tissue lysates were filtered through a 70 μm membrane. The cell suspension was centrifuged at 300×g and 4 °C for 5 min, after which the supernatant was fully aspirated, and the cells were washed and resuspended in PBS. Each sample comprised tissue from three mice. The study included three samples for Day 0, two for Day 7, and one for Day 30.

### Single cell RNA-sequencing

The single-cell suspension was loaded onto a Chromium Single-Cell Controller. Using the existing protocol, Single-cell 3’ library and gel bead Kit V3.1 (10xGenomics, 60318) and Chromium Single-cell A Chip Kit (10xGenomics, 60318) were used to generate single-cell gel beads (GEMs). Approximately 10,000 cells were added to each channel, with each library capturing up to 8,000 cells. Following cell capture and cleavage, reverse transcription reactions were performed to generate barcoded cDNA. Subsequently, barcoded RNA was produced by performing reverse transcription within each single GEMs. Each library was then sequenced using the NovaSeq platform (Illumina).

### Data processing for transcripts

**Fig. 1:**
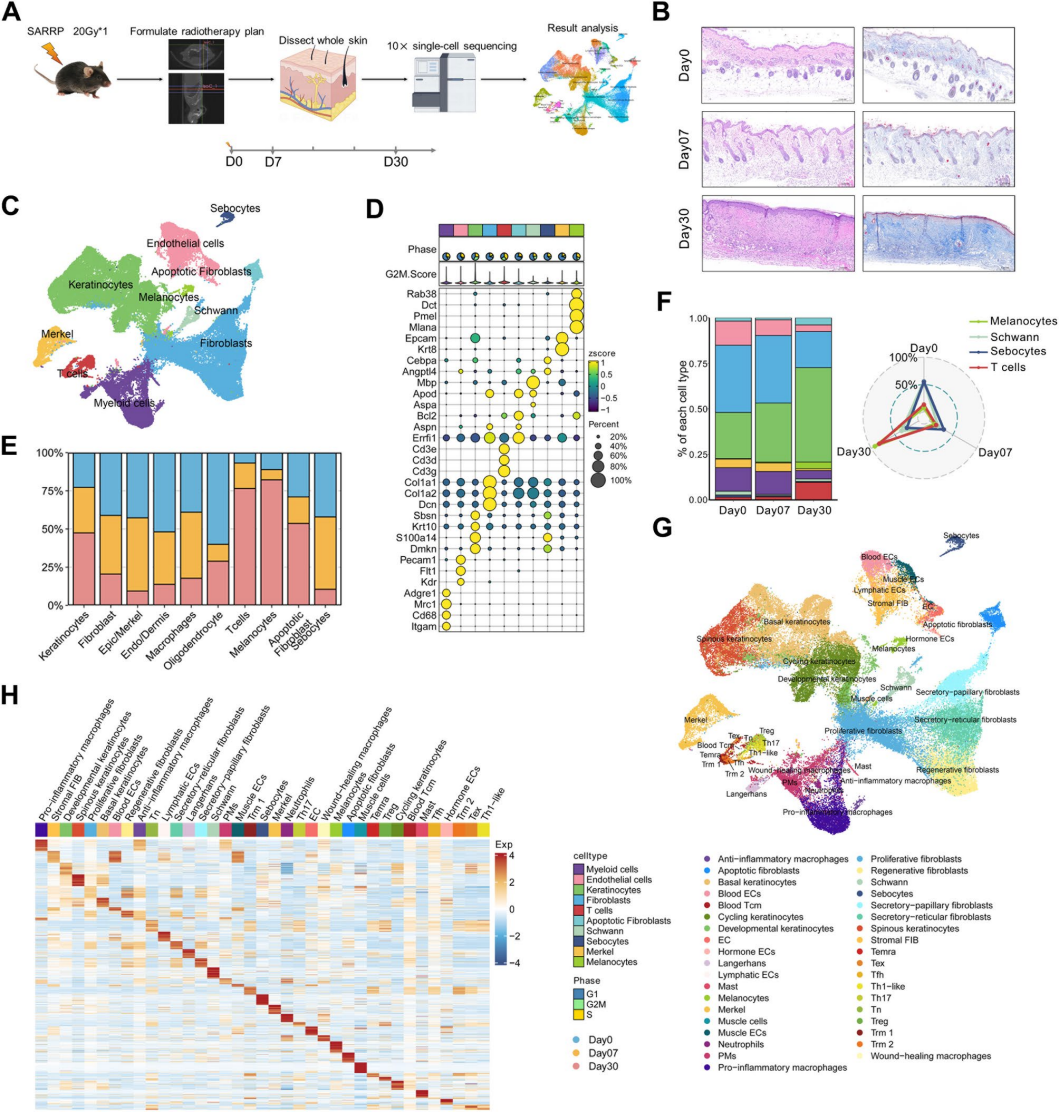
ScRNA-seq captures the cellular diversity in RISI. **A** Schematic overview and grouping information for single-cell RNA sequencing in this study. Skin tissues were isolated from the dorsal region of C57BL/6 mice at days 0, 7, and 30 post-irradiation, and subsequently processed for scRNA-seq using the 10X Genomics platform. Detailed information regarding the groups and treatment were provided in Table S1. **B** Representative images of skin samples stained with hematoxylin and eosin-stained (left) and masson (right) at days 0 (*n* = 6), 7 (*n* = 6), and 30 (*n* = 8) post-irradiation. Scale bar = 0.2 mm. **C** UMAP visualization of the total 71,412 cells harvested from mouse skin at days 0, 7, and 30 post-irradiation, categorized into 10 distinct clusters. **D** Dot plot depicting canonical markers and the proportion in G1/S/G2M state (up) for each cell type within the 10 defined clusters. The average expression levels were color-scaled and the dot size reflected the percentage of cells expressing the selected gene in each cell cluster. **E** Bar chart showing the relative contribution of the three states (days 0, 7, and 30 post-irradiation) to the total number of each cell type from scRNA-seq data. Non-repetitive sampling of 1,000 cells at each time point, including days 0, 7, and 30 post-irradiation, was conducted to eliminate the influence of cell number. **F** Bar chart (left) and Radar chart (right) showing the relative proportion of major cell types from scRNA-seq data, comparing various groups at days 0, 7, and 30 post-irradiation. **G** UMAP visualization of 71,412 cells from mouse skin at days 0, 7, and 30 post-irradiation, integrated into 37 assigned clusters. **H** Heatmap showing the top 50 differentially expressed genes across each of 37 subclusters. Day0: without radiotherapy. Day7: 7 days after radiotherapy. Day30: 30 days after radiotherapy

**Fig. 2:**
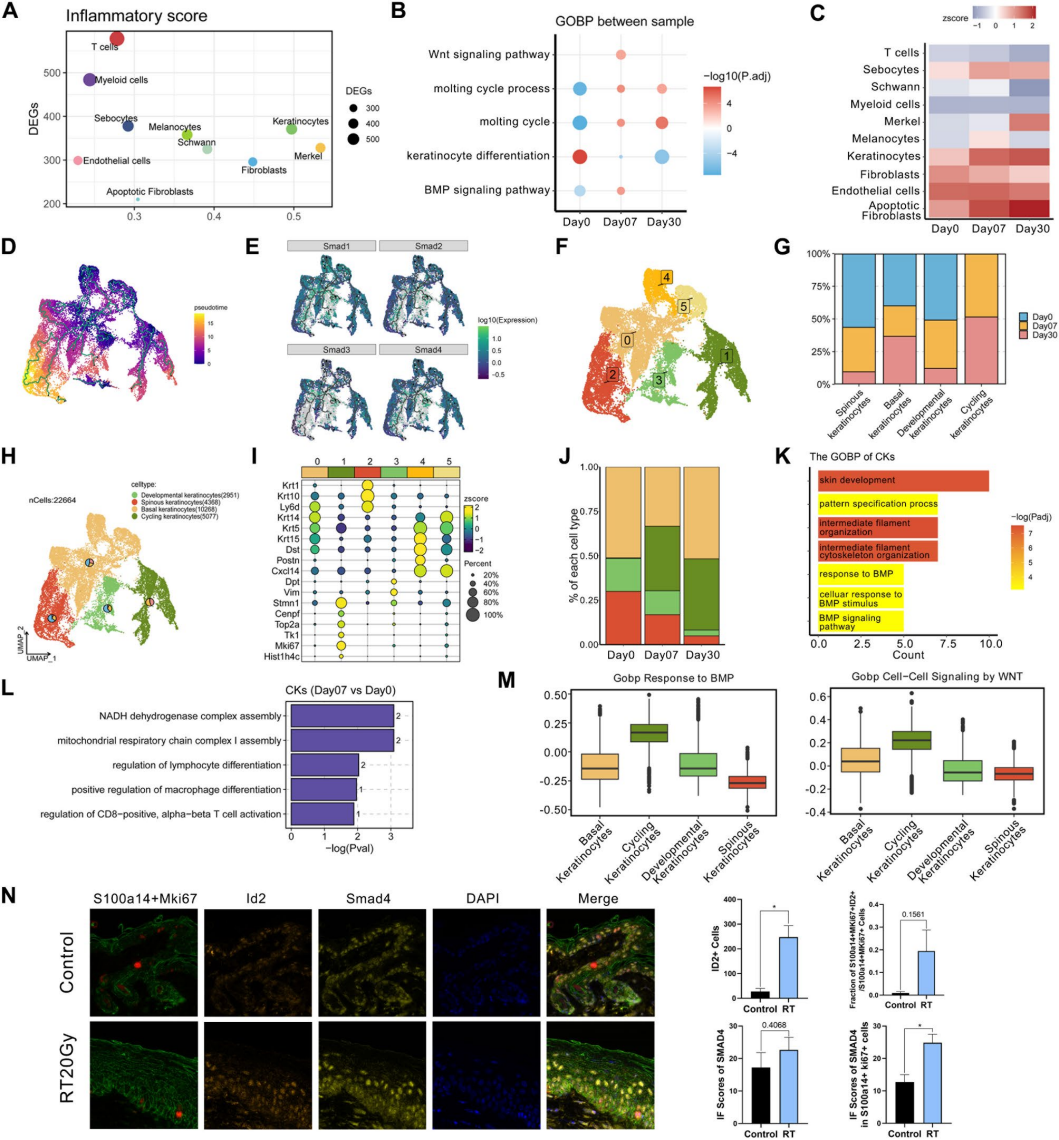
Characterization of KC subsets in RISI. **A** Dot plot depicting the number of DEGs and the inflammatory score across 10 subclusters. The inflammatory score signature was derived from the thirty-first pathway within the Hallmark gene sets, referred to as “HALLMARK_INFLAMMATORY_RESPONSE”. **B** Key GOBP pathways enriched keratinocytes (KCs) by DEGs of each group at days 0, 7, and 30 post-irradiation. **C** Heat map showing the expression of Smad1 among 10 distinct clusters at days 0, 7, and 30 post-irradiation. The color scheme was based on z-score distribution, ranging from − 1 (grey) to 2 (red). **D** Pseudotime analysis of different KC subtypes visualized through UMAP. As the evolutionary process progressed, the colors transitioned from purple to yellow. **E** Expression analysis of Smad family numbers 1–4 among different KC subtypes, presented through UMAP. As expression levels increased, the colors transitioned from navy blue to green. **F** UMAP visualization of 22,664 keratinocytes, color-coded into 6 distinct clusters. **G** Bar chart showing the relative contribution of the three states to the total number of each KC subcluster from scRNA-seq data. Non-repetitive sampling of 1,000 cells at each time point, including days 0, 7, and 30 post-irradiation, was conducted to eliminate the influence of cell number. **H** The relative proportions of cells at days 0, 7, and 30 (pie charts) and UMAP visualization of 22,664 keratinocytes harvest at days 0, 7, and 30 post-irradiation, categorized into 4 KC subclusters. **I** Dot plot depicting canonical markers for each KC subcluster. The average expression levels were color-scaled and the dot size reflected the percentage of cells expressing the selected gene in each cell cluster. **J** Bar chart showing the relative proportions of each KC subcluster from various groups at days 0, 7, and 30 post-irradiation. Each color represented a subpopulation, corresponding to the color of the subpopulation in Fig. 2H. **K** Key GOBP pathways enriched by upregulated DEGs in cycling keratinocytes, comparing to other KC subclusters. The redder the bar, the smaller the Padjust value. **L** Key GOBP pathways enriched by upregulated DEGs in cycling keratinocyte across days 7, comparing days 0. The numbers on the columns represented the quantity of DEGs associated with this pathway. **M** Box plot showing the single sample gene set enrichment analysis (ssGSEA) indicated the role of the 4 KC subtypes in “Gobp Response to BMP” and “Gobp Cell-Cell Signaling by WNT” pathways at day 7 post-irradiation. **N** Multiplexed immunofluorescence staining using S100a14 (green), Mki67 (red), Id2 (orange), Smad4 (yellow) and DAPI (blue) in mouse skin post-radiation therapy at days 0 (n = 6) and 30 (n = 8). The statistical column plots represent the Id2 positive fraction in indicated cells identified by canonical cell markers (S100a14 + Mki67, cycling keratinocytes) among groups (up) and the comparison of indicated Smad4 immunofluorescence scores among groups (down). Abbreviations: S100a14 = S100 Calcium Binding Protein A14; Mki67 = Marker of Proliferation Ki-67; Id2 = Inhibitor of DNA Binding 2; Smad4 = SMAD Family Member 4; DAPI = 4’,6-Diamidino-2’-phenylindole. Day0: without radiotherapy. Day7: 7 days after radiotherapy. Day30: 30 days after radiotherapy

The transcripts were mapped to the mm10 reference genome using Cell Ranger (5.0.0), generating a raw gene expression matrix for each scRNA-seq sample. The combined gene expression matrix was converted into a Seurat object using the R package *Seurat* (4.3.0). Cells expressing fewer than 200 or more than 10,000 genes were considered abnormal and excluded. Approximately 71,412 sequenced cells met quality control criteria (Figure S1A) and were used for downstream bioinformatics analysis, including three samples of 36,964 cells in the Con group (sample1: 13,823 cells, sample2: 10,704 cells, sample3: 12,437 cells), two samples of 23,469 cells in the Week group (sample1: 12,395 cells, sample2: 11,101 cells), and one sample of 10,952 cells in the Month group. Approximately 10,000 cells from each sample underwent single-cell RNA sequencing and were found to be normal [15]. When cell type was considered the independent variable and analyzing the relative contributions of the three states (days 0, 7, and 30 post-irradiation) to the total number of each cell type, non-repetitive sampling of 1,000 cells at each time point, including days 0, 7, and 30 post-irradiation, was conducted to eliminate the influence of cell number (Figs. 1E, 2G, 5D and 6B, and S6B). While analyzing the proportions of each cell type at days 0, 7, and 30 post-irradiation (such as Fig. 1F), the trend of proportion changes were consistent whether we used combined duplicate samples (Fig. 1F and S1F) or only one sample from each time point (Figure S1G and S1H). For the reason that the larger the sample size, the smaller the margin of error, we analyzed the proportion of each cell types at days 0, 7, and 30 post-irradiation without additional sampling (that means with the whole cells).

### Downstream analysis of transcripts

The column normalization and logarithmic transformation of gene expression matrix were carried out. To identify clusters of cells, a set of highly variable genes was first identified by packing the average expression of all genes into groups of uniform size and calculating the median dispersion in each group. Use the *NormalizeData* function to process integrated single-celled objects, select the normalized method LogNormalize. The R package *Harmony* was used to remove batch effects [16], after which a principal component analysis (PCA) and Uniform Manifold Approximation and Projection (UMAP) dimension reduction were performed. We used the JackStraw function in Seurat to identify significant PCA (1:30) and 10 cell clusters were identified using unsupervised clustering with resolution 0.5. The same procedure was followed for subcluster analysis of all cells. Keratinocytes were clustered using the top 10 PCA to generate 6 clusters with a resolution of 0.1. Fibroblasts were clustered using the top 10 PCA to generate 8 clusters with a resolution of 0.1. Endothelial cells were clustered using the top 10 PCA to generate 10 clusters with a resolution of 0.1. T cells were clustered using the top 10 PCA to generate 13 clusters with a resolution of 0.5. Myeloid cells were clustered using the top 10 PCA to generate 8 clusters with a resolution of 0.1.

### Cell-cycle analysis

The cycle fraction for each cell was calculated using the *CellCycleScoring* function, each cycle phase was assigned to metadata by set.ident = TRUE. Download the mouse_cell_cycle_genes file from github [17] reference genes associated with the cell cycle, including 42 G1/S and 52 G2M core genes (Table S2).

### Pseudotime analysis

We performed subcluster analysis of fibroblasts based on fibroblast specific markers. After that, the subgroup was transposed into the monocle analysis object. The custom functions were provided as follows. Monocle 2 (2.26.0) was used to simulate the pseudotime trajectories of these cells [18], using DDRTree’s dimensionality reduction algorithm, with other parameters as default. Difference analysis identified significantly altered genes dependent on pseudotime, considering all genes with q values below 0.1 as pseudotime-dependent. For the pseudo-timing of keratinocytes, Monocle 3 (1.0.0) was used to identify the pseudotime trajectories of the cells on the UMAP [19], employing default parameters.

The custom function:

# The seurat object was transposed into the monocle analysis object

seurat2cds = function(seuset){

seuset = AddMetaData(seuset, Idents(seuset), col.name="CellType”)

rawdata <- as(as.matrix(seuset@assays$RNA@counts),‘sparseMatrix’)

feature_ann<- data.frame(gene_id = rownames(rawdata), gene_short_name = rownames(rawdata))

rownames(feature_ann) <- rownames(rawdata)

result_fd <- new(“AnnotatedDataFrame”, data = feature_ann)

sample_ann <- seuset@meta.data

rownames(sample_ann) <- colnames(rawdata)

result_pd <- new(“AnnotatedDataFrame”, data = sample_ann)

result_cds <- newCellDataSet(rawdata, phenoData = result_pd, featureData = result_fd, expressionFamily = negbinomial.size())

return(result_cds)}

### RNA velocity analysis

The RNA velocity was determined from the spliced and unspliced transcript reads in the Cell Ranger output folder. Based on the *scVelo* Python package [20], the spliced and unspliced reads were computed, followed by basic preprocessing (gene selection and normalization), and the stochastic model was applied to estimate the RNA velocity. Subsequently, the velocity field was projected onto the UMAP space. The default parameters from the *velocyto* R package were used for the other parameters.

### Ligand and receptor interaction analysis

To understand intercellular communication, ligand and receptor interactions were inferred using the *Cellchat* (1.1.3) R package, based on the total population or subpopulation of interest at different time points [21]. Cellchat analysis, using default parameters, were performed to infer changes in the expression of specific pathways at different time points in this study. The annotation library was referred to CellChatDB.mouse. The results are mainly displayed using the netVisual_bubble and rankNet functions.

### Enrichment analysis

The ClusterProfiler (4.9.0) R package [22] was used to explore the biological themes associated with differentially expressed genes at various time points or between different cell types. Clusterprofiler is widely used for identifying the biological function of genes, including GO, KEGG, and others. For this study, biological processes with *p* values less than 0.05 in the GO database were primarily retained. The GSEA function was used to compare the expression level of the pathway between two samples, and used the default parameters. The results of GSEA were verified using the GOAT algorithm (Figure S3D-F) [23]. Single-sample enrichment scores were determined using the GSVA (1.46.0) R package [24], with the H.ARI.V7.4 gene bank used for enrichment scores.

### Inflammatory score

The inflammatory score signature utilized in our study was derived from the thirty-first pathway within the Hallmark gene sets, referred to as “HALLMARK_INFLAMMATORY_RESPONSE”. The Hallmark gene sets, which include 50 pathways of unique biological importance, were a vital and frequently utilized category within the MSigDB database. Comprehensive details regarding “HALLMARK_INFLAMMATORY_RESPONSE” could be accessed via the website [25].

### Multiplex immunofluorescence assay and analysis

Tissues fixed in 4% paraformaldehyde and embedded in paraffin were sectioned to a thickness of 4 μm. Tissue sections were deparaffinized using xylenes and rehydration through decreasing graded alcohols. AR6 buffer (Akoya Biosciences) was used for antigen retrieval in a microwave oven. Endogenous peroxidase was inactivated by incubation in 3% H2O2 for 10 min. Multiplex immunohistochemistry involved several rounds of staining, each consisting of a protein block using 1% BSA, followed by application of primary antibody and corresponding secondary horseradish peroxidase-conjugated antibody against mouse or rabbit immunoglobulins (Akoya Biosciences). The slides were then incubated with various Opal fluorophores (1:100) diluted in 1X Plus Amplification Diluent (Akoya Biosciences). Following tyramide signal amplification and covalent binding of the individual Opal fluorophores (Akoya Biosciences) to the targeted epitopes, the primary and secondary antibodies were removed via antigen retrieval, as mentioned earlier, and the subsequent cycle of immunostaining was initiated. The sequence of primary antibodies and Opal fluorophores were anti-S100A14/Opal 520, anti-Mki67/Opal 690, anti-ID2/Opal 620, anti-CD90/Opal 690, anti-Apcdd1/Opal 570, anti-MIP3 beta/Opal 520, anti-PROX1/Opal 570, anti-CCL21/Opal 620, anti-HMGB1/Opal 690. All slides were counterstained using spectral DAPI (Akoya Biosciences) and mounted with Anti-fade fluorescence mounting medium (ab104135, Abcam).

### Reverse transcription and qRT-PCR

RNA for qRT-PCR was extracted using TRIzol-based RNA extraction protocol. For each sample, 1 µg of total RNA was reverse-transcripted using the HiScript III RT SuperMix for qPCR (#R323-01, Vazyme, Nanjing, China). The generated cDNA was mixed with ChamQ Universal SYBR qPCR Master Mix (#Q711-02, Vazyme, Nanjing, China) and primers listed in Table S3. RT-qPCR reaction was performed using the qTOWER3 Real‑Time PCR Detection System (Analytik Jena GmbH, Jena, Germany). Target mRNAs were normalized against GAPDH, and data quantitation was performed using the comparative CT (ΔΔCT) method.

### Statistical analysis

All statistical analysis and graphing were conducted using R (version 4.3.0) and GraphPad Prism (version 8.0.1). Adjusted *p*-values above 0.05 were considered not significant, with the *p*-values in the figures indicated as follows: ∗*p* < 0.05, ∗∗*p* < 0.01, ∗∗∗*p* < 0.001, ∗∗∗∗*p* < 0.0001.

## Results

### ScRNA-seq analysis elucidates cellular composition in radiation dermatitis

To elucidate the cellular composition and transcriptional impact in radiodermatitis, we performed single-cell RNA sequencing (scRNA-seq) on mouse skin samples subjected to and spared from radiotherapy (RT) (Fig. 1A). To validate the clinical relevance of our model, we assessed the histopathological features of mouse skin samples, stained with hematoxylin and eosin, at 7 days and 30 days post-radiation exposure. Our analysis revealed pronounced morphological changes in RISI, such as keratinocyte hyperplasia and acanthosis in the epidermal layer, inflammatory cell infiltration, and alterations in hair follicles and capillaries in the dermal layer. Masson staining further revealed collagen deposition and the extent of fibrosis across different experimental groups (Fig. 1B).

To improve our capacity to identify infrequent cell clusters, we employed a rigorous quality control workflow to integrate and analyze data from samples collected at different time points. This involved exclusion of low-quality cells, culminating in a dataset of 71,412 cells (Table S4). Utilizing Uniform Manifold Approximation and Projection (UMAP) for visualization, we identified 32 distinct cell clusters (Figure S1C), which were further classified into 10 major cell types (Fig. 1C and S1D) based on canonical cell markers (such as *Col1a1* and *Col1a2* for fibroblasts, *Pecam1* for endothelial cells, and *Krt10* for keratinocytes) (Fig. 1D and S1E). A notable cluster, termed “apoptotic fibroblast”, exhibited high expression of fibroblast-related marker *ASPN* [26], as well as apoptosis-related marker *Bcl-2*, yet lacked *Thy-1* (*CD90*), a recognized fibroblast marker [27]. Importantly, all 10 clusters comprised cells from both control and post-radiation (day 7 and 30) mouse skin biopsies (Fig. 1E). Cell composition analysis revealed an increased proportion of keratinocytes (KCs) and T cells at day 7 post-irradiation, with further amplification by day 30 (Fig. 1F and S1F). This implies a potential pathogenic role of these cell types in RISI. For deeper insights, we subclustered keratinocytes, fibroblasts, endothelial cells, and immune cells, identifying 37 subgroups (Fig. 1G and S1I). We conducted differential gene expression analysis to identify cell-type-specific marker genes, visualizing the top 50 ranked genes for each cell type in a heatmap (Fig. 1H). In conclusion, our extensive skin injury atlas offers an in-depth understanding of the cellular dynamics and transcriptomic alterations induced by radiation, shedding light on underlying mechanisms.

### Role of cycling keratinocytes with activated BMP in wound healing and fibrosis in RISI

The epidermis, primarily composed of stratified layers of keratinocytes, serves as the skin’s outermost protective barrier and a biosensor to the external environment [3]. Keratinocytes are also recognized as key contributors to RISI pathogenesis, particularly in initiating inflammation [28]. Our study noted substantial changes in KCs following RT, with their proportion among sequenced cells rising from 25.3 to 51.8% at 30 days post-RT (Figure S1F). This aligns with the known fact that high radiotherapy doses can induce heightened mitotic activity in the basal keratinocyte layer [8]. This rapid regeneration of new cells, coupled with slower old cell shedding, may lead to skin thickening and scaling, known as dry desquamation. We delved into this process by examining KC sub-populations located in the upper right corner of the coordinate system, using a “HALLMARK_INFLAMMATORY_RESPONSE” gene set to compute an “inflammatory score”, thereby assessing both inflammatory response and differentially expressed genes (DEGs) (Fig. 2A).

To gain a deeper understanding of the potential functions and active signaling pathways of KCs post-RT, we conducted a GO functional enrichment analysis (Fig. 2B). The analysis unveiled an upregulation in molting cycle process and related signaling pathways. Furthermore, the activation of the WNT signaling pathway and the involvement of BMP pathways suggested that KCs played a dual role in wound healing and mediating radiation-induced fibrosis. Given that *BMPs* belong to the transforming growth factor beta (TGF-β) superfamily and *TGF-β*’s role in fibrosis induction via *Smad3* activation, we investigated the Smad family expression. The gene expression analysis revealed an upregulation of *Smad* in KCs post-irradiation (Fig. 2C and S2A), aligning with the gene ontology biological process (GOBP) analysis outcomes. Furthermore, the *Smad* expression and the trajectory analysis illustrated a time-dependent increase in *Smad* levels (Fig. 2D and E). Collectively, these findings suggested a gradual development of fibrosis following RT.

Subsequent subclustering analysis of KCs revealed 6 distinct subclusters (Fig. 2F), with KC1 primarily consisting of KCs from samples subjected to RT (Fig. 2G and S2B). Utilizing a combination of scRNA-seq and specific markers in the radiation dermatitis, we classified KC0, KC4, and KC5 as basal keratinocytes, KC1 as cycling keratinocytes, and KC2 as spinous keratinocytes (Fig. 2H and I). Notably, KC3 emerged as a novel sub-population of KCs, characterized by unique markers distinct from the classical keratin subgroups, and demonstrated a significant decrease during radiodermatitis (Fig. 2J). These KC3 cells, identified as developmental keratinocytes, displayed activated G2M/S states and enhanced extracellular matrix (ECM)-related signaling pathways (Figure S2C and S2D). The relative proportions of these subtypes varied markedly as radiodermatitis progressed (Fig. 2J), aligning with the activation of the molting cycle-related pathways (Fig. 2B). This variation also corroborated findings from a previous study suggesting that highly proliferative and well-oxygenated cells are the most radiosensitive in the body [3].

Single-cell trajectory analysis of developmental keratinocytes identified an actively proliferating sub-population, marked by G2M/S state activation and the expression of stemness-associated genes, such as *CD34* and *Ly6a* (Figure S2C and S2E) [29]. These cells possess a remarkable ability to migrate downwards and differentiate into basal cells, eventually contributing to the formation of hair follicles. They can also migrate upwards and differentiate into various types of epidermal cells [30]. Notably, radiotherapy specifically targeted and eliminated the more radiosensitive developmental keratinocytes. Furthermore, the GO analysis revealed enriched BMP signaling pathways in cycling keratinocytes (Fig. 2K), suggesting their role in fibrosis development. Beyond regulating lymphocyte and macrophage differentiation (Fig. 2L and S2F), the substantial enrichment in Wnt signaling pathways and increased count of cycling keratinocytes suggested their potential role in radiodermatitis development, wound healing, and skin integrity maintenance (Fig. 2M and S2G). To investigate the distribution of pro-inflammatory and pro-fibrotic cycling keratinocytes in injured skin, we analyzed immunofluorescence (IF) staining, bulk RNA sequencing data from 8 mouse skin samples, scRNA sequencing data from rat samples, and scRNA sequencing of human dataset. Following RT, the inflammatory scores in the skin tissue increased, accompanied by a significant upregulation of ID2 and SMAD4 expression (Fig. 2N and S2H). Further analysis indicated a substantial contribution from the cycling keratinocytes subcluster (Fig. 2N, S2I and S2J). Collectively, our data provided a more comprehensive characterization of cycling keratinocytes with enriched Wnt and BMP signaling pathways, revealing a distinct transcriptional profile likely impacting wound healing and fibrosis in RISI.

### Identification of terminally differentiated secretory-papillary fibroblasts with immune cell recruitment capacity in RISI

The dermis, located beneath the epidermis, plays a crucial role in maintaining skin’s structural integrity. It primarily comprises connective tissue, synthesized by dermal fibroblast [3]. In pre-RT skin, fibroblasts constitute the largest cell group, accounting for 36.9% (Figure S1F). Irradiation significantly impacted fibroblast abundance (Fig. 1F), suggesting their potential involvement in radiodermatitis progression. Analysis of UMAP and RNA velocity data revealed a strong correlation between fibroblasts and Schwann cells, particularly in their G2M/S states (Fig. 1C, S3A, and S3B). It is plausible that the Schwann cell cluster originated from fibroblasts. We thus integrated data from Schwann cells, apoptotic fibroblasts, and fibroblasts for further analysis.

**Fig. 3:**
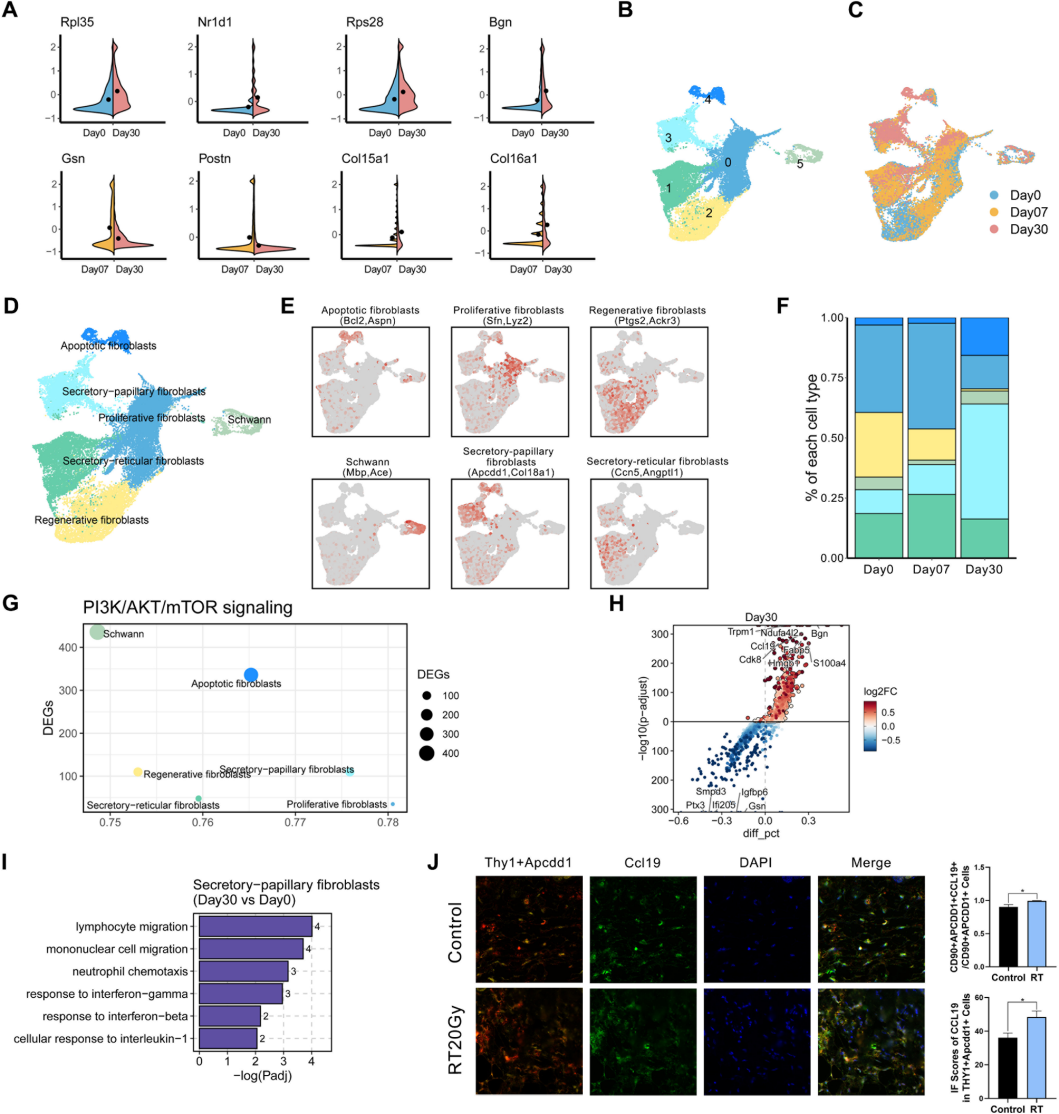
Characterization of Fib subsets in RISI. **A** Violin plot showing DEGs in Fibs in control/30 days post-RT group comparison (up) as well as 7 days post-RT/30 days post-RT group comparison (down). The black point in each image represented the mean value for the corresponding group, calculated using the summarySE function. **B** UMAP visualization of 26,971 fibroblasts, color-coded into 6 clusters. **C** UMAP showing Fib composition from various groups at days 0, 7, and 30 post-irradiation. **D** UMAP visualization of 26,971 fibroblasts harvest at days 0, 7, and 30 post-irradiation, categorized into 6 Fib subclusters. **E** UMAP plots of canonical markers for Fib subclusters in the skin samples. Each subcluster was defined using two canonical markers, and the expression levels of both markers were mapped onto the UMAP plots. **F** Bar chart showing the relative proportions of each Fib subcluster from various groups at days 0, 7, and 30 post-irradiation. Each color represented a subpopulation, corresponding to the color of the subpopulation in Fig. 3D. **G** Number of DEGs of each Fib subcluster relative to the PI3K/AKT/mTOR signaling. The “PI3K/AKT/mTOR signaling” signature was derived from the Hallmark gene sets. **H** Volcano plot showing DEGs of secretory-papillary fibroblasts across day 30, comparing day 0. The genes shown in red indicate upregulation, while those in blue represent downregulation at day 30 post-irradiation, comparing day 0. **I** Key GOBP pathways enriched by upregulated DEGs of secretory-papillary fibroblasts, comparing days 30 and 0. The numbers on the columns represented the quantity of DEGs associated with this pathway. **J** Multiplexed immunofluorescence staining for Thy1 (red), Apcdd1 (yellow), Ccl19 (green), and DAPI (blue) in mouse skins treated with radiation therapy at days 0 (n = 6) and 30 (n = 8). The statistical column plots represent the Ccl19 positive fraction in indicated cells identified with canonical cell markers (Thy1 + Apcdd1, secretory-papillary fibroblasts) among groups (up) and the comparison of indicated Ccl19 immunofluorescence scores among groups (down). Abbreviations: Thy1 = Thy-1 Cell Surface Antigen; Apcdd1 = APC Down-Regulated 1; Ccl19 = C-C Motif Chemokine Ligand 19; DAPI = 4’,6-Diamidino-2’-phenylindole. Day0: without radiotherapy. Day7: 7 days after radiotherapy. Day30: 30 days after radiotherapy

To elucidate the effects of radiotherapy on fibroblast sub-populations and their role in radiation-induced skin reactions, we analyzed the significantly altered genes. Besides genes associated with ribosomes and mitochondria (such as *Rpl35*, *Rps28*, and *mt-Co1*), a considerable number of genes related to metabolism, inflammation, and immune response (such as *Nr1d1* and *Bgn*) were upregulated following RT (Fig. 3A and S3C). This suggested that radiation exposure not only affected fibroblast metabolism but also influenced immune sub-populations, culminating in an inflammatory response within fibroblasts. Considering temporal changes, we analyzed differentially expressed genes in samples collected at 7 and 30 days post-RT. Relative to day 7, the day 30 samples exhibited increased expression of collagen-associated genes, such as *Col15a1* and *Col16a1*. Conversely, genes related to inflammation and extracellular matrix (ECM), such as *Gsn* and *Postn*, were downregulated in the day 30 samples (Fig. 3A and S3C). This observation was supported by Gene Set Enrichment Analysis (GSEA) and GOAT, revealing significant enrichment in ECM components and focal adhesion pathways (Figure S3D-F).

Fibroblasts, segregated from the aggregate cell population, were classified into eight distinct sub-populations (Fig. 3B and C). Notably, subsets Fib0, Fib6, and Fib7 were distinguished by an upregulated expression of collagen regulatory genes (*Sfn*) post-irradiation (Figure S3G) [31], indicative of their proliferative nature, underscored by active G2M/S states. On the other hand, Fib1 was characterized by an elevated expression of secretory-reticular fibroblast markers *Ccn5* and *Angptl1* [32]. Furthermore, highly expressed *Ackr3*, *Fn1*, and *Ptgs2*, known for their significant roles in inflammation, tissue restoration, and wound healing, were identified in Fib2 (Figure S3H). This observation suggested that Fib2 represented a sub-population of fibroblasts closely associated with tissue regeneration. Fib3 was distinguished by an elevated expression of secretory-papillary fibroblast markers *Apcdd1* and *Col18a1*. Fib4, classified as apoptotic fibroblasts, showed increased expression of apoptotic genes, notably *Bcl-2*. Fib5, characterized by the expression of Schwann cell markers (such as *Mbp*) (Fig. 3D and E), played a role in nerve repair. Additionally, the composition of fibroblast cells underwent rapid and significant changes following RT (Fig. 3F).

We further investigated the role of these fibroblast sub-populations in the pathogenesis of RISI. “PI3K/AKT/mTOR signaling” referred to Hallmark, which mediates inflammation [33], was prominently associated with secretory- papillary fibroblasts (Fig. 3G). Previous studies have indicated that secretory-papillary fibroblasts are involved in ECM reorganization, collagen synthesis, and fibrosis [34]. However, in our dataset, we observed their significant immune activation functions, as evidenced by the upregulated genes (Fig. 3H and S3I) and enriched pathways (Fig. 3I and S3J). The marked activation of immune cells (lymphocytes, monocytes, and neutrophils) and cytokine responses (IL-1 and IFN), highlighted the role of pro-inflammatory secretory-papillary fibroblasts (Figure S3K), with a terminal differentiation phenotype (Figure S3L), in IFN response, immune cell recruitment, and the inflammatory response in RISI. IF analysis of the inflammatory factor CCL19 highlighted the pro-inflammatory role of secretory-papillary fibroblasts in skin tissue following irradiation (Fig. 3J), which was further validated using scRNA sequencing data from both rat and human samples (Figure S3M-O). Considering their remarkable changes, we speculated that these common secretory-papillary fibroblasts exhibited unique functional activity and played a crucial role in the injury response.

### Potential role of fibs and KCs subclusters interactions in restoration via epithelial-mesenchymal transition (EMT)

The notable changes in the quantity and functional attributes of Fibs and KCs highlighted their crucial roles in radiodermatitis progression. The activation of the BMP signaling pathway in KCs suggested that KC sub-populations might contribute to the fibrosis process, recognized as a delayed side effect of radiotherapy. However, the development of chronic dermatitis and skin fibrosis has traditionally been ascribed to the activity of dermal fibroblasts [8]. Considering these insights, we posited that fibroblasts and keratinocytes might synergistically influence the evolution of radiation dermatitis, marked by extensive intercellular interactions.

We employed a heatmap to visualize the correlation of gene expression between fibroblast sub-populations and the novel Developmental keratinocyte sub-population, which led us to focus on the Proliferative fibroblasts (Figure S4A). Pearson correlation analysis revealed a robust relationship between Developmental keratinocytes and Proliferative fibroblasts, evidenced by a high R-value (0.88) (Figure S4B). Additionally, these two sub-populations were located closest to each other in the UMAP plot (Fig. 1G). Cell-cell communication between these sub-populations was analyzed using CellChat to elucidate their biological interplay (Figure S4C and S4D). Notably, Proliferative fibroblasts exhibited elevated levels of EMT-associated ligands, such as collagens (encoded by *COL1A1*, *COL1A2*, *COL6A1*, *COL6A2*, *COL6A3*), fibronectin 1 (encoded by *FN1*), and Thy-1 (encoded by *THY1*) [35]. Developmental keratinocytes expressed receptors capable of recognizing various ligands, facilitating intercellular interaction and communication. The EMT signaling pathway is critically involved in the wound healing process [36]. Consequently, Proliferative fibroblasts may promote the EMT in Developmental keratinocytes through cell-cell communication via multiple signaling pathways in RISI. Additionally, the expression analysis of *Col1a2* revealed that it was not only expressed in Proliferative fibroblasts but also across almost all fibroblast sub-populations (Figure S4E), indicating their potential role in regulating KCs as a ligand. *Sdc*’s influence extends beyond affecting the function of keratinocytes (like reduced migration and altered matrix), it also enhances TGF-β signaling [37]. Collectively, the activated Fibs post-RT may induce the pro-fibrotic functions in KCs through the secretory of inflammatory factor and collagen, contributing to the wound healing process.

Besides interactions between Developmental keratinocytes and Proliferative fibroblasts, extensive communications among other sub-populations were noted (Figure S4F). To investigate the mechanism of radiodermatitis, we specifically focused on the clusters with significant post-RT increases. This included interactions between Apoptotic fibroblasts and basal keratinocytes, Apoptotic fibroblasts and developmental keratinocytes, and regenerative fibroblasts and developmental keratinocytes, among others. These interactions offered critical insights into the pathogenesis of radiodermatitis. In comparison to the ligands related to EMT and TGF-β, the upregulation of Wnt and Bmp family members in Fibs and KCs subclusters implicated their roles in in promoting wound healing and fibrosis in RISI (Figure S4G). Consequently, we hypothesized that the ligand-receptor pairs involved in the processes of tissue restoration and fibrosis vary across different subpopulations.

### The role of pro-inflammatory lymphatic ECs in mediating leukocyte trafficking in RISI pathogenesis

With advancements in cancer therapeutics leading to extended patient survival, the significance of delayed radiation effects, particularly those caused by vascular endothelial injury, becomes more apparent [38]. Furthermore, it is well-established that a single dose of radiation exceeding 10 Gy can result in severe vascular damage [39]. This is demonstrated in our data by the marked decrease in radiation-induced endothelial cells (ECs) on day 7, with a continued decline noted on day 30 (Fig. 1F).

**Fig. 4:**
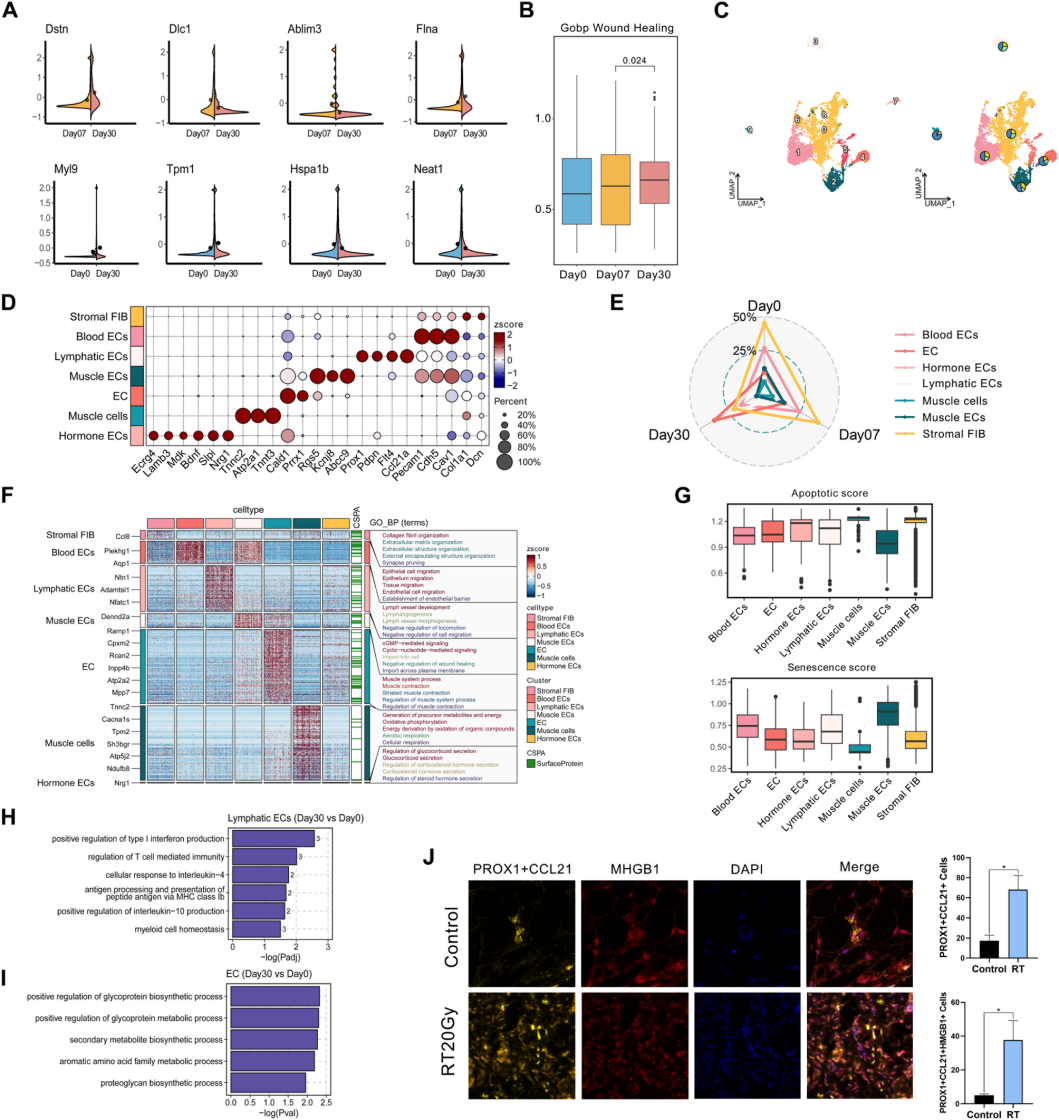
Characterization of EC subsets in RISI. **A** Violin plot showing DEGs of ECs in 7 days post-RT/30 days post-RT group comparison (up) as well as control/30 days post-RT group comparison (down). The black point in each image represented the mean value for the corresponding group, calculated using the summarySE function. **B** Box plot showing the “Wound Healing” score of ECs at days 0, 7, and 30 post-irradiation by ssGSEA enrichment analysis. **C** UMAP plots of 7,319 endothelial cells (ECs), color-coded into 10 subclusters (left); The relative proportions of cells at days 0, 7, and 30 (pie charts) and UMAP visualization of 7,319 endothelial cells harvest at days 0, 7, and 30 post-irradiation, categorized into 7 EC subclusters (right). **D** Dot plot depicting canonical markers for each EC subcluster. The average expression levels were color-scaled and the dot size reflected the percentage of cells expressing the selected gene in each cell cluster. **E** Radar chart showing the relative proportions of EC subsets from various groups at days 0, 7, and 30 post-irradiation. **F** Heat map showing the top most differentially expressed genes (left) and representative GO terms enriched by upregulated DEGs in each EC subcluster (right). **G** Box plot showing the “Apoptotic score” (up) and “Senescence score” (down) among each EC subset by ssGSEA enrichment analysis. **H** Key GOBP pathways enriched by upregulated DEGs of Lymphatic ECs across days 30, comparing days 0. The numbers on the columns represented the quantity of DEGs associated with this pathway. No significantly enriched pathways were identified at Day 7 (consistent with Fig. 4I), therefore, Day 7 data are not shown. **I** Key GOBP pathways enriched by upregulated DEGs of ECs across days 30, comparing days 0. The numbers on the columns represented the quantity of DEGs associated with this pathway. **J** Multiplexed immunofluorescence staining for PROX1 (yellow), CCL21 (orange), HMGB1 (red), and DAPI (blue) in mouse skins treated with radiation therapy at days 0 (n = 6) and 30 (n = 8). The statistical column plots represent the change of double or triple positive cells (PROX1 + CCL21, Lymphatic ECs) among groups. Abbreviations: PROX1 = Prospero Homeobox 1; CCL21 = C-C Motif Chemokine Ligand 21; HMGB1 = High Mobility Group Box 1; DAPI = 4’,6-Diamidino-2’-phenylindole. Day0: without radiotherapy. Day7: 7 days after radiotherapy. Day30: 30 days after radiotherapy

Our study identified significant changes in the expression of genes involved in cell migration and angiogenesis in ECs following RT. Notably, there was an upregulation of *Ablim3* [40] and *Dlc1* [41] during the initial stage of skin remodeling and an increase in *Flna* and *Dstn* expression in the later stages. A consolidated analysis of both RT groups showed comparable outcomes, highlighting changes in the expression of myosin-related gene *Myl9* [42], vascularization-related gene *Hspa1b* [43], and *Neat1* [44] (Fig. 4A and S5A). Collectively, these observations imply that radiation’s impact on ECs played a role in modulating angiogenesis and neovascularization. To gain further insight into the potential functions and active signaling pathways of specific EC subpopulations, we conducted ssGSEA enrichment analysis among various groups on day 0, 7, and 30 post-irradiation (Fig. 4B). The enhanced pathway of wound healing suggests a reparative role played by ECs in the context of RISI.

We isolated 7,319 ECs from the total cell population and categorized them into 10 distinct subpopulations (Fig. 4C). During vascular remodeling, we observed variations in the relative distribution of these subpopulations. Subsequently, we examined the expression profiles of these subclusters. EC0 and EC8, characterized by robust expression of matrix-associated markers (*Col1a1* and *Dcn*), were identified as stromal fibroblasts (Fig. 4D and E and S5B). EC1 and EC6, expressing endothelial marker genes (*Pecam1*, *Cdh5*) [10, 45], and high levels of cell membrane structure genes like *Cav1*, were classified as Blood ECs. Furthermore, E2, showing elevated expression of genes (*Rgs5*, *Kcnj8*, *Abcc9*) and associated pathways (Fig. 4F and S5C), was designated as Muscle ECs. EC3, expressing *Prox1*, *Pdpn*, and *Flt4* genes (Figure S5B), key promoters of lymphatic vessel formation, and co-expression of *Ccl21a*, was identified. Following previous research [10], EC4 and EC5 were annotated as double negative stromal ECs. Notably, the transcription factors *Prrx1*, pivotal in preserving endothelial integrity and maintaining vascular homeostasis, was highly expressed in EC4 and EC5, suggesting their involvement in restoration processes. It was worth noting that the smallest EC subpopulation (EC7) exhibited elevated expression of growth factor genes, such as *Bdnf* and *Nrg1*, suggesting a possible role in growth regulation. Among these subpopulations, only SMC9 displayed expression of myofilament genes (*Tnnc2*, *Lmod2*, *Tnnt3*), linked to smooth muscle cell function. Collectively, these findings suggest a transformation of endothelial cells, primarily of blood vascular endothelial and fibroblastic phenotype, into lymph-like and double negative stromal cell-like cells in remodeling lesions (Fig. 4E). Subsequent GO analysis revealed enrichment of second-messenger-mediated signaling pathways in nearly all categories of EC sub-populations (Fig. 4F). Furthermore, there was specific activation of lymph vessel development in EC3 and pronounced expression of pathways associated with glucocorticosteroid and hormone process in EC7 (Fig. 4F). Considering that acute radiation exposure induces EC apoptosis, while late chronic effects result in EC senescence and fibrotic changes [46], we hypothesized that the muscle cells within the EC subcluster were most affected during both early and late phases, as reflected by the apoptotic and senescence scores (Fig. 4G).

We then investigated the functional changes in EC subsets in RISI. While both ECs and lymphatic ECs showed an increase in number, further analysis indicated more pronounced upregulation of pathways related to inflammation, cytokine signaling (IL and IFN), cell recruitment, and antigen processing and presentation (MHC) in lymphatic ECs in RISI. Conversely, ECs exhibited a more evident enhancement in metabolic pathways (Fig. 4H and I). Besides an inflammatory milieu (Figure S5D), we observed an accumulation of lymphatic ECs and their heightened pro-inflammatory activity post-RT, as determined through IF analysis (Fig. 4J). Furthermore, the role of lymphatic ECs was validated using scRNA sequencing data from both rat and human samples (Figure S5E and S5F). These findings imply that lymphatic ECs, characterized by their pro-inflammatory properties, may be the specific subsets actively contributing to RISI pathogenesis by facilitating leukocyte migration and promoting inflammation.

### Functional parallels and heterogeneity in activated immune cell sub-populations during radiation dermatitis

Considering the role of ECs, KCs, and Fibs in activating immune cells during radiation-induced inflammation [3], which contributes to the second phase of radiation-induced skin reactions [46], it is important to note that the cascading amplification of immune cell-driven inflammatory responses plays a significant role in radiodermatitis pathogenesis. Moreover, the unpredictable emergence of successive inflammatory waves, occurring weeks to years post-radiation exposure, further complicates the management of radiation burns. Our study focused on the two predominant immune cell subpopulations, T cells and myeloid cells, to investigate the inflammatory response in radiation dermatitis. Given the divergent infiltration patterns of these two clusters post- RT, we extracted and analyzed them independently.

**Fig. 5:**
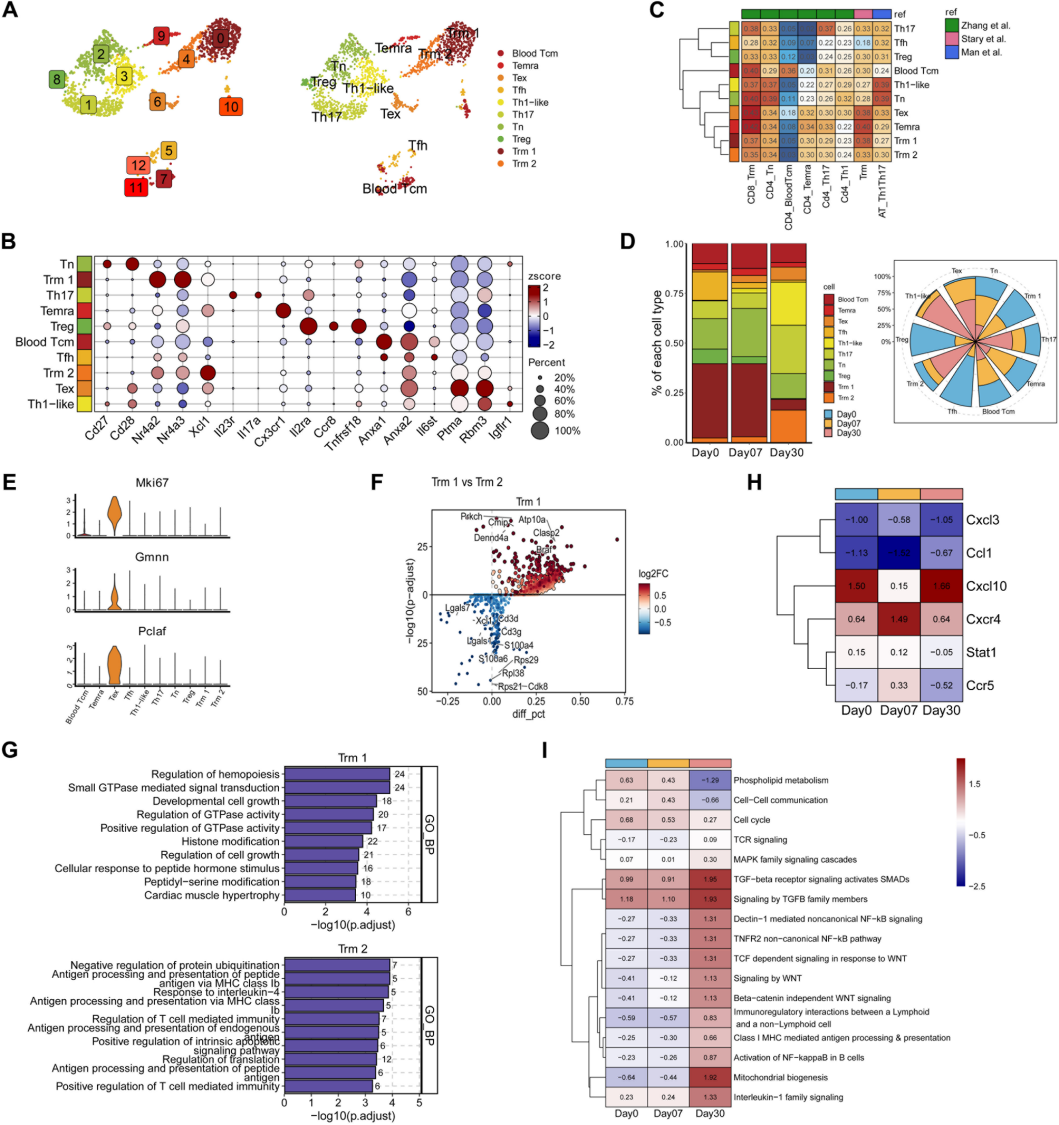
Characterization of T cell subsets in RISI. **A** UMAP plot of 2,029 T cells, color-coded into 13 subclusters (left); UMAP visualization of 2.029 T cells harvest at days 0, 7, and 30 post-irradiation, categorized into 10 T cell subclusters (right). **B** Dot plot depicting canonical markers for each T cell subcluster. The average expression levels were color-scaled and the dot size reflected the percentage of cells expressing the selected gene in each cell cluster. **C** Heatmap showing the correlation between each T cell subcluster in our study with T cell subcluster in the reference studies. **D** Bar chart showing the relative proportions of each T cell subcluster from various groups at days 0, 7, and 30 post-irradiation (left); Rose plot showing the relative contribution of the three states to the total number of each T cell subcluster from scRNA-seq data (right). Non-repetitive sampling of 1,000 cells at each time point, including days 0, 7, and 30 post-irradiation, was conducted to eliminate the influence of cell number. **E** Expression of the Mki67, Gmnn, and Pclaf mRNA among 10 different subtypes of T cells, visualized in violin plot. **F** Volcano plots showing DEGs in Trm1/Trm2 comparison. The DEGs up-regulated in Trm1 were color-coded with red and down-regulated with blue. **G** Key GOBP pathways enriched by upregulated DEGs of Trm1 (up) and Trm2 (down). The numbers on the columns represented the quantity of DEGs associated with this pathway. **H** Heatmap showing the differential expression chemokines among various groups at days 0, 7, and 30 post-irradiation. **I** Heatmap showing Key pathways of ssGSEA enrichment analysis among various groups at days 0, 7, and 30 post-irradiation. Day0: without radiotherapy. Day7: 7 days after radiotherapy. Day30: 30 days after radiotherapy

Typically, the quantity of T lymphocytes in human skin is about twice that found in the peripheral blood [47]. To delve deeper into their skin-related effects, we clustered T cells into 10 cell types based on their canonical lineage markers, as referenced in previous studies (Fig. 5A and B) [48]. The accuracy of this clustering was further validated by additional studies (Fig. 5C) [48]. Among these, tissue-resident memory T (Trm) cells, which play a crucial role in skin immunity [28], constitute the majority, with smaller proportions of other cell types (Fig. 5D). Post-irradiation, there was an upsurge in Tex, Th1-like, Th17, and Trm2 cells. These significant changes suggested their potential pathogenic roles and the contribution to immune imbalance during radiation-induced skin inflammation (Fig. 5D). Tex cells exhibited cell cycle markers (*Mki67*, *Gmnn*, *Pclaf*) (Fig. 5E), signifying robust, while the increase in other subpopulations could be due to their recruitment and infiltration into the skin.

The contrasting patterns between Trm1 and Trm2 highlighted their distinct roles in RISI. To gain further insights into the cellular characteristics of these Trm cell sub-populations, we conducted a marker gene analysis (Fig. 5F). Our analysis revealed that Trm2 expressed elevated levels of *Cd3d* and *Cd3e*, critical genes in T cell development and signal transduction, suggesting an activated T cell phenotype. Additionally, this subset also manifested high expression of ribosome-related genes (*Rps21*, *Rpl38*). Conversely, Trm1 expressed *CMIP* and *DENND4A* genes, along with enriched function-associated pathways (Fig. 5G). This provided crucial insights into the distinct molecular profiles and potential functions of these Trm cell sub-populations in RISI.

To investigate the role of T cell subsets in RISI, we performed an analysis of inflammatory molecules and pathways. The analysis revealed the expression of tissue retention molecules (*CXCR4*) [49] and various chemokines (*CCL1*, *CXCL3*) in our datasets (Fig. 5H). Interestingly, these genes were upregulated in the skin post- irradiation, indicating that cells newly infiltrating the skin may induce residency marker genes and adopt a resident phenotype. Notably, in RISI, ssGSEA revealed that pathways associated with inflammation, such as NF-KB signaling, transforming growth factor β (TGF-β) signaling, IL-1 signaling, and antigen presenting pathways, were significantly upregulated, particularly 30 days post-RT (Fig. 5I). The temporal gene regulation analysis suggested that a T cell subset overexpressed tissue retention markers, chemokines, and receptors critical for the survival of specific population of cells [50], enabling their persistence following radiotherapy.

**Fig. 6:**
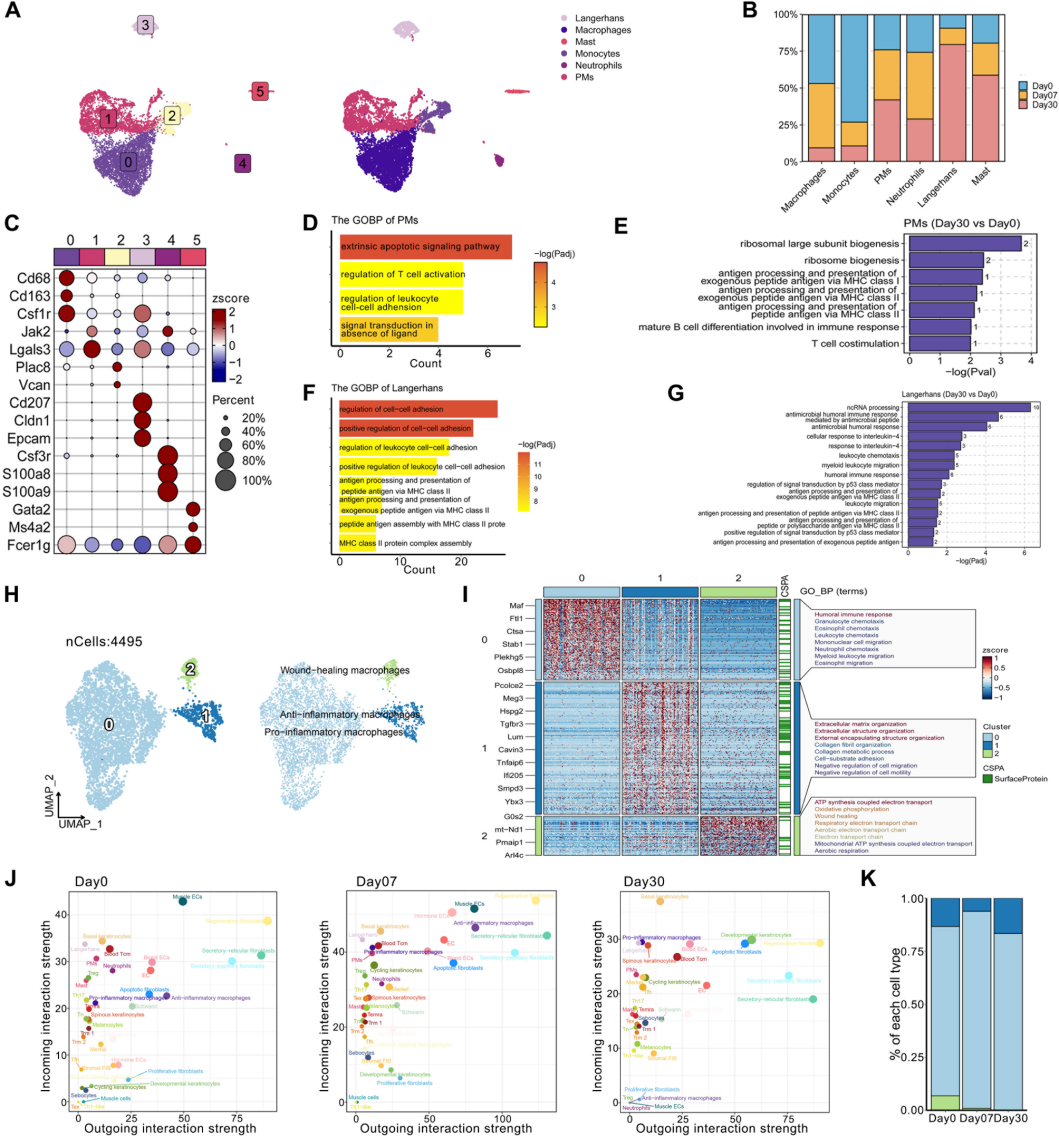
Characterization of myeloid cell subsets in RISI. **A** UMAP plot of 8,238 myeloid cells, color-coded into 6 subclusters (left); UMAP visualization of 8,238 myeloid cells harvest at days 0, 7, and 30 post-irradiation, categorized into 6 myeloid cell subclusters (right). **B** Bar chart showing the relative proportions of each myeloid cell subcluster from various groups at days 0, 7, and 30 post-irradiation from scRNA-seq data. Non-repetitive sampling of 1,000 cells at each time point, including days 0, 7, and 30 post-irradiation, was conducted to eliminate the influence of cell number. **C** Dot plot depicting canonical markers for each myeloid cell subcluster. Each color representing the 6 subpopulations corresponded to the colors of the subpopulations shown in Fig. 6A. The average expression levels were color-scaled and the dot size reflected the percentage of cells expressing the selected gene in each cell cluster. **D** Key GOBP pathways enriched by upregulated DEGs of PMs. The redder the bar, the smaller the Padjust value. **E** Key GOBP pathways enriched by upregulated DEGs of PMs across days 30, comparing days 0. The numbers on the columns represented the quantity of DEGs associated with this pathway. No significantly enriched pathways were identified at Day 7 (consistent with Fig. 6G), therefore, Day 7 data are not shown. **F** Key GOBP pathways enriched by upregulated DEGs of Langerhans. The redder the bar, the smaller the Padjust value. **G** Key GOBP pathways enriched by upregulated DEGs of Langerhans across days 30, comparing days 0. The numbers on the columns represented the quantity of DEGs associated with this pathway. **H** UMAP plot of 4,495 monocytes and macrophages, color-coded into 3 subclusters (left); UMAP visualization of 3 monocyte and macrophage subclusters (right). **I** Heat map showing the top most differentially expressed genes (left) and Key GO pathway enriched by upregulated DEGs in each monocyte and macrophage subcluster (right). Each color representing the 3 subpopulations corresponded to the colors of the subpopulations shown in Fig. 6H. **J** NetAnalysis_signalingRole_scatter analysis of incoming and outgoing interaction strength in the total subpopulation of our dataset among groups at days 0, 7, and 30 post-irradiation. **K** Bar chart showing the relative proportions of each monocyte and macrophage subcluster from various groups at days 0, 7, and 30 post-irradiation. Each color represented a subpopulation, corresponding to the color of the subpopulation in Fig. 6H. Day0: without radiotherapy. Day7: 7 days after radiotherapy. Day30: 30 days after radiotherapy

Myeloid cells, characterized by their diversity and dynamism, play crucial roles in various biological processes. In our RISI samples, we identified various myeloid cell subtypes, including Langerhans cells (LCs), macrophages, mast cells, monocytes, neutrophils, and proliferative myeloid cells (PMs) (Fig. 6A-C).

The top DEGs in PMs, such as *Lgals3* and *Jak2*, were predominantly linked to cell proliferation (Figure S6C). Besides their rapid proliferation, GOBP analysis revealed that PMs also manifested activated inflammatory pathways and a heightened ability to recruit immune cells relative to other myeloid cell types (Fig. 6D). Furthermore, this recruitment ability was enhanced following irradiation (Fig. 6E). Another notable myeloid cell subtype, Langerhans cells, also exhibited a significant change in quantity. As professional antigen-presenting cells responsible for activating T cells in immune responses, Langerhans cells were associated with the regulation of MHC class II molecules and leukocytes, consistent with their established function (Fig. 6F) [28]. Interestingly, post-irradiation enrichment pathway analysis revealed that Langerhans cells, in addition to their traditional functions, stimulated pathways related to inflammation and cytokine responses (Fig. 6G). Together, these findings indicated that irradiation substantially activates inflammatory reactions in the expanded myeloid cell subclusters, highlighting their collaborative involvement in RISI.

Monocytes and macrophages were further categorized into three major populations based on their functions: pro-inflammatory macrophages, anti-inflammatory macrophages, and wound-healing macrophages (Fig. 6H) [28]. GO analysis revealed that Mφ0 was involved in the regulation of leukocyte chemotaxis, implying a potential pro-inflammatory role (Fig. 6I). Furthermore, the analysis of cell interaction strengths demonstrated an increased signaling activity in pro-inflammatory macrophages, highlighting their critical role within the microenvironment 7 and 30 days post-RT compared to the control group (Fig. 6J). Mφ1 was highly involved in pathways related to ECM organization, alongside its role in collagen metabolism (Fig. 6I). Previous studies have recognized the ECM as a medium with anti-inflammatory properties, capable of downregulating inflammation-related genes and factors in macrophages, thereby mitigating localized inflammatory responses [51]. Mφ2 exhibited elevated expression of pathways related to wound-healing, cell migration, and adhesion (Fig. 6I). Their enriched signatures indicated their distinct roles in inflammation. The relative proportion of macrophage subtypes and their interaction strengths suggested that Mφ0 with a pro-inflammatory phenotype might contribute to the pathogenesis of radiodermatitis (Fig. 6K). These findings highlighted the limitations of traditional marker panels in accurately capturing the functional states of macrophages in vivo.

Overall, these findings illustrated the presence of diverse populations of T cells and myeloid cells in remodeled skin models. The differential gene expression signatures and enriched signaling pathway results observed in T cells and myeloid cells provided insights into the transcriptional and functional discrepancies between the innate and adaptive immune responses during radiation-induced skin reactions. Nonetheless, there were also notable similarities between myeloid cells and T cells. GOBP analysis using hallmark gene sets revealed a parallel enrichment of signaling pathways in both myeloid cells and T cells during skin remodeling. Notably, both myeloid cells and T cells showed enrichment in inflammation and chemokine regulation pathways, highlighting their importance in the immune response during skin remodeling. Other pathways, including IL-1 signaling, positive regulation of chemokine (C-X-C motif) ligand 2 production, and cell chemotaxis, among others, were also identified as key drivers in KC, Fib, or EC subclusters (Figure S6D-S6F).

### Development of a regulatory network among KCs, Fibs, and ECs subclusters with immune cells in RISI

**Fig. 7:**
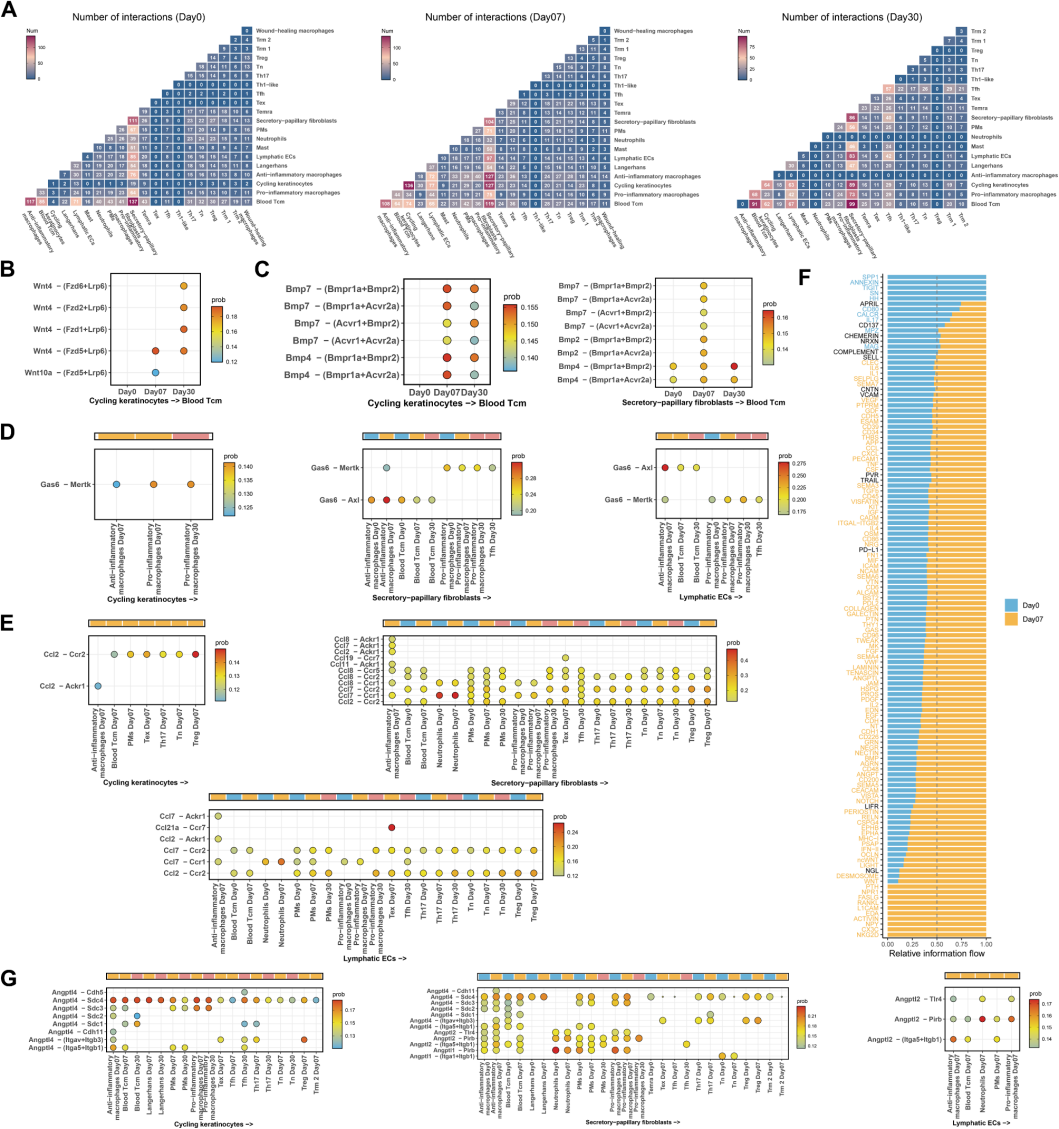
Intercellular communication among KCs, Fibs, and ECs subclusters with immune cells in RISI. **A** Heatmap showing the interaction intensity among cycling keratinocytes, secretory-papillary fibroblasts, and lymphatic endothelial cells with immune cell subclusters at days 0 (left), 7 (center), and 30 (right) post-irradiation in RISI. **B** The interactions of cycling keratinocytes with Blood Tcm via Wnt interaction pairs in mouse skin samples at days 0, 7, and 30 post-irradiation. In the context of receptor-ligand relationships between the two subpopulations, the arrow pointed from the ligand to the receptor (as shown below the figure). For receptor-ligand relationships involving genes, the ligand was typically indicated on the left side of the dash, while the receptor was positioned on the right of the dash, sometimes enclosed within parentheses (as depicted in the vertical coordinates). **C** The interactions of cycling keratinocytes and Secretory-papillary fibroblasts with Blood Tcm via Bmp interaction pairs in mouse skin samples at days 0, 7, and 30 post-irradiation. **D** The interactions of cycling keratinocytes, Secretory-papillary fibroblasts, and Lymphatic ECs with immune cells via Gas6 interaction pairs in mouse skin samples at days 0, 7, and 30 post-irradiation. The color above the figure represented various time points of the samples. Same meaning as the previous colors, day0 (blue), day7 (orange), and day30 (pink). **E** The interactions of cycling keratinocytes, Secretory-papillary fibroblasts, and Lymphatic ECs with immune cells via Ccl interaction pairs in mouse skin samples at days 0, 7, and 30 post-irradiation. **F** The relative information flow of secreting signals in the control and 7 days post-irradiation groups. **G** The interactions of cycling keratinocytes, Secretory-papillary fibroblasts, and Lymphatic ECs with immune cells via Angptl interaction pairs in mouse skin samples at days 0, 7, and 30 post-irradiation. Day0: without radiotherapy. Day7: 7 days after radiotherapy. Day30: 30 days after radiotherapy

Considering the crucial role of immune cells in RISI, we conducted intercellular communication analysis to explore their involvement. The results showed an escalation in ligand-receptor pair interactions among cycling keratinocytes, secretory-papillary fibroblasts, lymphatic endothelial cells, and immune cells with the progression of radiodermatitis (Fig. 7A). Previous studies have documented these resident cells, including KCs, Fibs, and ECs, react to radiation by activating early response genes and proteins, encompassing growth factors, chemokines, and cytokines. These molecules attract inflammatory cells from epidermal and dermal layers, and circulation [3], contributing to effective host defense and tissue homeostasis in RISI [28, 46]. We focused on these specific subclusters, delving into their intricate interaction signaling with immune cells. We observed an upregulation of the Wnt pathway in cycling keratinocytes post-RT, driven predominantly by Wnt4 and Wnt10a (Fig. 7B and S7A). Conversely, BMP signaling in other subclusters showed an initial increase but later declined in interactions with specific immune cells, including Blood Tcm, anti-inflammatory macrophages, and Trm2, with a noticeable deficiency observed in lymphatic ECs (Fig. 7C and S7B). Furthermore, we found that the upregulated gene associated with senescence, Gas6, could bind to ligands from diverse immune cells (Fig. 7D), aligning with earlier findings on irradiation effects [10, 52]. In the context of skin diseases, studies have demonstrated that the chemokine *CXCL8*, produced by M2 macrophages, can engage with endothelial cells expressing *ACKR1*. This interaction plays a crucial role in the recruitment of immune cells to sites of inflammation [53]. In our study, we witnessed heightened interactions involving chemokines (including *Cxcl1*, *Cxcl2*, *Cxcl10*, *Ccl7*, and *Ccl2*) and *Ackr1* among KCs, Fibs, ECs subclusters, and immune cells following RT. These vigorous interactions a significant involvement of these subclusters in recruiting leukocytes to the skin in RISI (Fig. 7E and S7C). Overall, these specific receptor-ligand communications further emphasized the critical contributions of cycling keratinocytes, secretory-papillary fibroblasts, and lymphatic endothelial cells in RISI development.

To explore the key signaling pathways influencing radiodermatitis, we compared each pathway in non-irradiated samples and post-irradiation samples, using the concept of information flow. This approach enabled us to quantify the probability of communication between each pair of cell groups (Fig. 7F and S7D). We observed notable activation of several pathways, including MIF, ANGPTL, and MK, in the irradiation groups. Intriguingly, although both MIF and ANGPTL signaling increased post-RT, the emergence of the pro-angiogenic gene *Angptl4* was particularly notable in cycling keratinocytes post-irradiation, which exhibited interactions with nearly all immune cells. In contrast, no marked changes were observed in MIF signaling (Fig. 7G and S7E). These findings suggested that the augmented ANGPTL signaling was significantly influenced by enhanced interactions with immune cells, whereas the changes in MIF signaling may stem from different underlying factors. Collectively, our analysis highlighted substantial alterations in cell-cell communication networks in radiodermatitis and identified key signaling changes that might contribute to RISI pathogenesis.

## Discussion

Given its primary exposure and high vulnerability to radiation, the skin is susceptible to radiation dermatitis during cancer treatments and following nuclear incidents [1]. Meanwhile, the prevalence of radiodermatitis is increasing due to the extensive use of radiotherapy, substantially impacting patients’ quality of life [54]. Effective management and control of radiation-induced reactions necessitate a thorough understanding of the complex, heterogeneous microenvironment. In this regard, scRNA-seq has emerged as an unbiased comprehensive technique for analyzing cells from tissue and peripheral blood [55, 56]. We presented a comprehensive single-cell analysis of RISI in both its acute and chronic phases using preclinical models. To ensure that the dose we selected was comparable to clinical doses for human skin, we applied the concept of biologically effective dose (BED) to confirm that the 20 Gy dose in our model accurately represents the conventional dose used in patients. The detailed calculation is as follows: generally, acute tissue responses are characterized by higher α/β values compared to late tissue responses, though specific values can vary across species. A prior review compared α/β values among different species, finding notable similarities between human skin and pig skin, while results for mouse skin differed under comparable conditions [57]. Based on the previous analysis estimating the α/β value of 10 Gy [57] and utilizing our 20 Gy dose, the BED (BED = n×d×(1 + d/(α/β)) was 60 Gy (*n* = 1, d = 20 Gy, and α/β = 10 Gy). The equivalent delivered dose in humans, where the α/β ratio is 7.5 Gy [58], was 17.79 Gy. In summary, our dose of 20 Gy in mice is approximately equivalent to 17.79 Gy in humans, placing it within the recommended dose range for clinical applications [59]. Thus, we opted for a dose of 20 Gy for our research. Despite variations in radiation types—from X-rays to electron beams—the most commonly used time points are Day 7, Day 14, and Day 30 post-irradiation in significant recent pre-clinical studies investigating RISI [5, 60, 61]. Since acute radiation effects (e.g., erythema, desquamation, ulceration) typically appear around Day 5 and peak at Day 14 [61], while chronic effects have been studied around Day 21 [62], we conducted a preliminary experiment and found significant fibrosis in the mice’s skin by one month. Considering both the radiation type and the species (C57BL/6 mice), we designated one month to represent the chronic irradiation response and one week to represent the acute response in humans. We identified a total of 37 cell clusters across three different time points in this study. Through integrated analysis of scRNA-seq data, we identified various cell subtypes activated during inflammation, including cycling keratinocytes, secretory-papillary fibroblasts, and lymphatic endothelial cells. These cell types exhibited high cytokine gene expression, and their pathways were notably enriched in leukocyte recruitment process. Moreover, they actively interacted with immune cells via a variety of chemokines, adhesion molecules, and inflammatory molecules. To the best of our knowledge, this is the inaugural study to comprehensively characterize the immune cell-based cellular landscape and its dynamic functional evolution in RISI. Our study provides a detailed insight into the critical changes and interactions occurring at the single-cell level across various stages of RISI. These findings shed light on the underlying mechanisms of RISI development and may provide valuable guidance for clinical prevention and treatment strategies.

Our dataset al.so revealed the presence of rarer cells, such as melanocytes and Merkel cells. Although the number of melanocytes was initially low, it significantly increased at Day 30 post-irradiation (Fig. 1F and S1F). GO functional enrichment analysis revealed an upregulation of biosynthetic and metabolic processes at Day 7 post-irradiation (Figure S1J). In addition to pathways related to melanin biosynthesis, energy-related pathways were also upregulated at Day 30 post-irradiation (Figure S1K). These findings suggest that the overall function of melanocytes did not change significantly after radiotherapy. In addition, as a gentle touch receptor in the skin, the Merkel cell-neurite complex plays an important role in the skin [63]. GOBP enrichment analysis of Merkel cells indicated activation of pathways associated with ATP response, DNA-binding transcription factors, and ion transport at Day 7 post-irradiation (Figure S1L), while pathways related to cell development, differentiation, and the molting cycle were upregulated at Day 30 post-irradiation (Figure S1M). Interestingly, despite the activation of pathways related to the molting cycle, the number of Merkel cells decreased following radiotherapy (Fig. 1F and S1F).

It is well-documented that the Smad family plays a significant role in mediating fibrosis. Interestingly, studies on mice lacking *Smad3* have demonstrated expedited wound healing accompanied by a diminished local inflammatory response [64]. In contrast to the well-established pro-fibrotic role of fibroblasts, notably *Smad4*’s involvement in Radiation-Induced Heart Fibrosis [65], the role of keratinocytes in fibrosis has not been extensively studied. Our observations revealed that keratinocytes with elevated *Smad* expression showed higher inflammatory scores, enhanced molting cycling, and activated Wnt and BMP pathways. These findings indicated that keratinocytes, alongside fibroblasts, could play a significant role in mediating fibrosis in RISI. Notably, the increased number of cycling keratinocytes with heightened BMP signaling activity was linked to upregulated Wnt signaling and leukocyte differentiation pathways, indicating their role in fibrosis. Moreover, among the three key pro-inflammatory subclusters (including cycling keratinocytes, secretory-papillary fibroblasts, and lymphatic endothelial cells), we observed exclusive activation of the Wnt pathway in cycling keratinocytes following irradiation. This implies a distinct role for this cell subtype in response to RT. Overall, our findings strongly indicate that cycling keratinocytes, post-irradiation, played a crucial role as sentinel cells in lymphocyte and macrophage differentiation, activation of specific T cells, and chemokine biosynthesis, and mediating wound healing and fibrosis via Wnt and BMP/Smad pathways.

Fibroblasts, present in various organs, are instrumental in the synthesis and maintenance of the ECM components. In the wound healing process, the ECM acts as a double-edged sword, either facilitating wound healing or contributing to fibrosis, depending on its levels [66]. Our study noted a reduction in fibroblast count 30 days post-RT, likely due to the matrix remodeling in skin wound repair, which involves fibroblast apoptosis from 21 days to 1 year post-injury [67]. Typically, human skin wound healing is driven mainly by dermal fibroblasts from the reticular dermis, as opposed to the papillary dermis [68]. Research suggests that reticular fibroblasts have the potential to differentiate into papillary fibroblasts [69], a hypothesis that was supported by our RNA velocity results (Figure S3B). Notably, in our single-cell dataset, secretory-papillary fibroblasts also played a crucial role in the wound healing process. This indicated that the evolution from secretory-reticular fibroblasts to secretory-papillary fibroblasts is a dynamic and integral aspect of wound healing. Consequently, it would be inaccurate to define a single major sub-population as the sole mediator of the wound healing. Furthermore, in line with the characteristic markers and the evolutionary pattern observed in the pathogenic subcluster of thyroid-associated ophthalmopathy (TAO) [70], apoptotic fibroblasts, the only Thy-1(CD90)^−^ subcluster, possessed a strong capacity to differentiate into adipocytes [71]. These findings, coupled with GO analysis results (Figure S3P), suggested that apoptotic fibroblasts may be another critical subtype in RISI, alongside the earlier-described secretory-papillary fibroblasts. Our study proposes that wound healing is likely a dynamic process, and a specific subcluster of fibroblasts with Thy-1 (CD90)^−^ features, capable of adipocyte differentiation, might also play a pivotal role in diverse injury responses.

Previous research has mainly focused on the detrimental effects of substantial damage to ECs in disease development [72]. However, our data uncovered a distinct aspect of injury response following irradiation, particularly in the subtype of the Lymphatic ECs. We observed a considerable accumulation of chemokines, such as *Ccl21a*, which mediate inflammation. Additionally, inflammation pathways, including cytokine signaling, cell recruitment, and antigen processing and presentation, were activated in lymphatic ECs. Intriguingly, without the interaction between the Bmp ligand and immune cells, the upregulated subpopulation may have a limited role in fibrosis. These findings provided substantial evidence that, beyond the reduced number of endothelial cells, certain subtypes with a pro-inflammatory phenotype were also involved in RISI pathology.

Cutaneous dendritic cells (DCs), such as epidermal Langerhans cells (LCs), interstitial/dermal dendritic cells (DDCs), and plasmacytoid DCs (pDCs), play a crucial role in immune responses, especially under pathological conditions [73]. LCs, serving as a critical immunological barrier against the external factors, are typically maintained locally in the skin under normal conditions. However, inflammatory changes can lead to their replacement by blood-borne LC progenitors [74]. LCs are renowned for their strong for their potent antigen processing and presentation capabilities. Besides an increase in their numbers, LCs also exhibited a response to cytokines following RT. Furthermore, radiation can selectively induce mast cell mediators, subsequently modulating the release of chemokines from dermal fibroblasts [75]. This effect notably impacts the recruitment of inflammatory cells into irradiated skin. While the quantity of mast cells in our dataset was limited, their role in recruiting immune cells (Figure S6G) and potential contribution to inflammation amplification in RISI should not be underestimated. In summary, our single-cell dataset not only confirmed mast cell presence in RISI and also highlighted the potent inflammatory effects of LCs beyond their traditional roles.

In conclusion, our study conducted a comprehensive transcriptional characterization of the changes occurring in the skin pre- and post-radiation therapy at a high-dimensional single-cell level in RISI. The results of our research delineated the potential pathogenic roles of proliferating cycling keratinocytes, secretory-papillary fibroblasts, and lymphatic endothelial cells in triggering or amplifying RISI. These cell types activate pathways associated with inflammation, secrete chemokines, upregulate critical genes, and regulate immune cells through ligand-receptor interactions. Targeting these interactions could potentially alleviate complications associated with RISI. We believe that our findings will contribute to a better understanding of the subclusters and molecular mechanisms involved in radiodermatitis, serving as a valuable database for future investigations into the pathogenesis of RISI.

While this study offers valuable insights into the skin tissue conditions in RISI, it is important to recognize several potential limitations. Firstly, despite the clinical advantages of diverse radiation methods like proton and heavy ion therapy over conventional photon therapy [76], our study was limited to mice with RISI exposed to X-ray radiation. Mice exposed to proton or heavy ion radiation were not included in our study. Future investigations should incorporate mice or individuals exposed to a broader range of radiation methods to provide a more comprehensive understanding of RISI. Secondly, the isolation and processing procedures utilized for scRNA-seq of skin cells may potentially introduce biases in cell recovery. Although integrating multiple samples aided in reducing this potential bias, employing supplementary techniques, such as spatial transcriptomics technology, would further strengthen the reliability of these findings. Furthermore, while murine models provide numerous benefits and serve as a valuable complement to clinical biopsy samples, it is important to exercise caution when interpreting conclusions based solely on murine data until they have been confirmed and validated using human samples or cells. In addition, our study focused solely on the skin and did not include the subcutaneous compartment, which is indeed a valuable area for further investigation. Moreover, we did not adequately consider the different types of radiation and the associated injuries in various skin layers. Future research could compare the effects of different radiation types, such as X-rays, electron beams, and ^60^Co γ radiation, among others. Additionally, we did not discuss the impact of varying radiation doses in our study. Future studies could explore the effects of different radiation doses, along with various time points.

## Electronic supplementary material

Below is the link to the electronic supplementary material.

Supplementary Material 1

## Data Availability

The original 3′-end scRNA-seq data based on bam format have been deposited at Genome Sequence Archive (GSA) and are available to the public on the date of publication (GSA: CRA014692).

## Abbreviations

RISI: Radiation induced skin injury
RT: Radiotherapy
scRNA-seq: Single-cell RNA sequencing
RT-PCR: Reverse transcription polymerase chain reaction
KCs: Keratinocytes
Fibs: Fibroblasts
ECs: Endothelial cells
ROS: Reactive oxygen species
PCA: Principal component analysis
UMAP: Uniform manifold approximation and projection
DEGs: Differentially expressed genes
TGF-β: Transforming growth factor beta
GOBP: Gene ontology biological process
ECM: Extracellular matrix
IF: Immunofluorescence
GSEA: Gene set enrichment analysis
Trm: Tissue-resident memory T
LCs: Langerhans cells
PMs: Proliferative myeloid cells
DCs: Dendritic cells
