# Supplementary material for: Molecular profiling of skin cells identifies distinct cellular signatures in radiation-induced skin injury across various stages in the murine dataset
Source: Exp Hematol Oncol. 2025 Feb 25;14:18. doi: 10.1186/s40164-025-00596-w (PMC11852861; doi:10.1186/s40164-025-00596-w)
Supplement: Supplementary file 1 — Supplementary Material 1 [file 40164_2025_596_MOESM1_ESM.docx]

**Additional information**

**
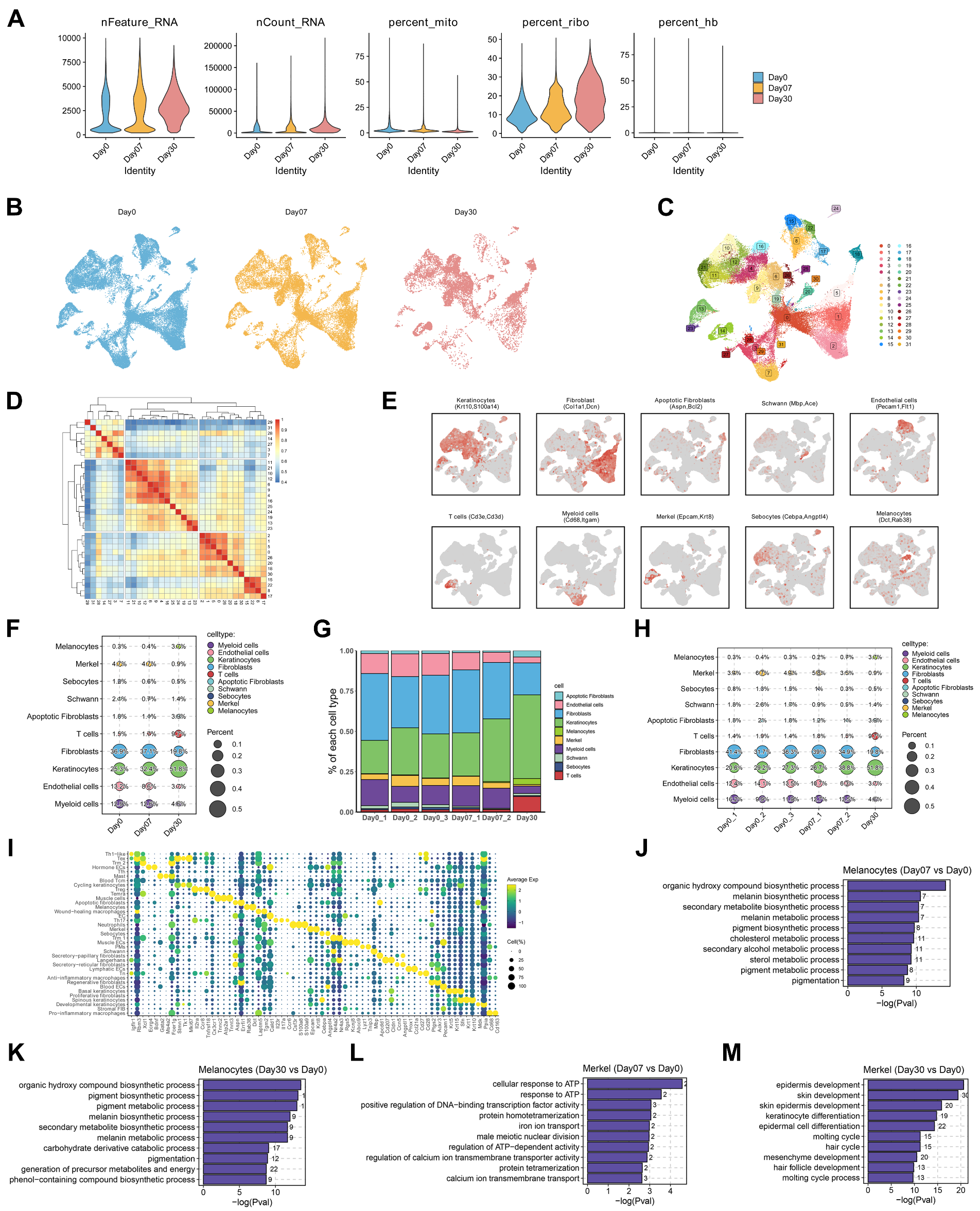
**

**Supplementary Fig. S1. Canonical markers for each cell type. Corresponding to Figures 1. A.** The percentages of “nFeature_RNA”, “nColunt_RNA”, “percent_mito” (mitochondria genes), “percent_ribo” (ribosome genes), and “percent_hb” (hemoglobin genes) following gene filtration at specified cutoff values. **B.** UMAP showing cluster composition according to cell origin from various groups at days 0, 7, and 30 post-irradiation. **C.** UMAP visualization of 71,412 cells, color-coded into 32 cluster. **D.** Heatmap showing aggregations of similar clusters. **E.** UMAP plots of canonical markers for the 10 assigned clusters in the skin samples. Each subcluster was defined using two canonical markers, and the expression levels of both markers were mapped onto the UMAP plots. **F.** Dot plot showing the relative proportion of major cell types from various groups at days 0, 7, and 30 post-irradiation. **G.** Bar chart showing the relative proportion of major cell types from each sample derived from scRNA-seq data, comparing various groups at Day0_1, Day0_2, Day0_3, Day07_1, Day07_2, and Day30 post-irradiation. **H.** Dot plot showing the relative proportion of major cell types from various groups at Day0_1, Day0_2, Day0_3, Day07_1, Day07_2, and Day30 post-irradiation. **I.** Dot plot depicting canonical markers for each cell type of the 37 assigned clusters. The average expression levels were color-scaled and the dot size reflected the percentage of cells expressing the selected gene in each cell cluster. **J.** Key GOBP pathways enriched by upregulated DEGs in Melanocytes across days 7, comparing days 0. **K.** Key GOBP pathways enriched by upregulated DEGs in Melanocytes across days 30, comparing days 0. **L.** Key GOBP pathways enriched by upregulated DEGs in Merkel cells across days 7, comparing days 0. **M.** Key GOBP pathways enriched by upregulated DEGs in Merkel cells across days 30, comparing days 0. The numbers on the columns represented the quantity of DEGs associated with this pathway. Day0: without radiotherapy. Day7: 7 days after radiotherapy. Day30: 30 days after radiotherapy.

**
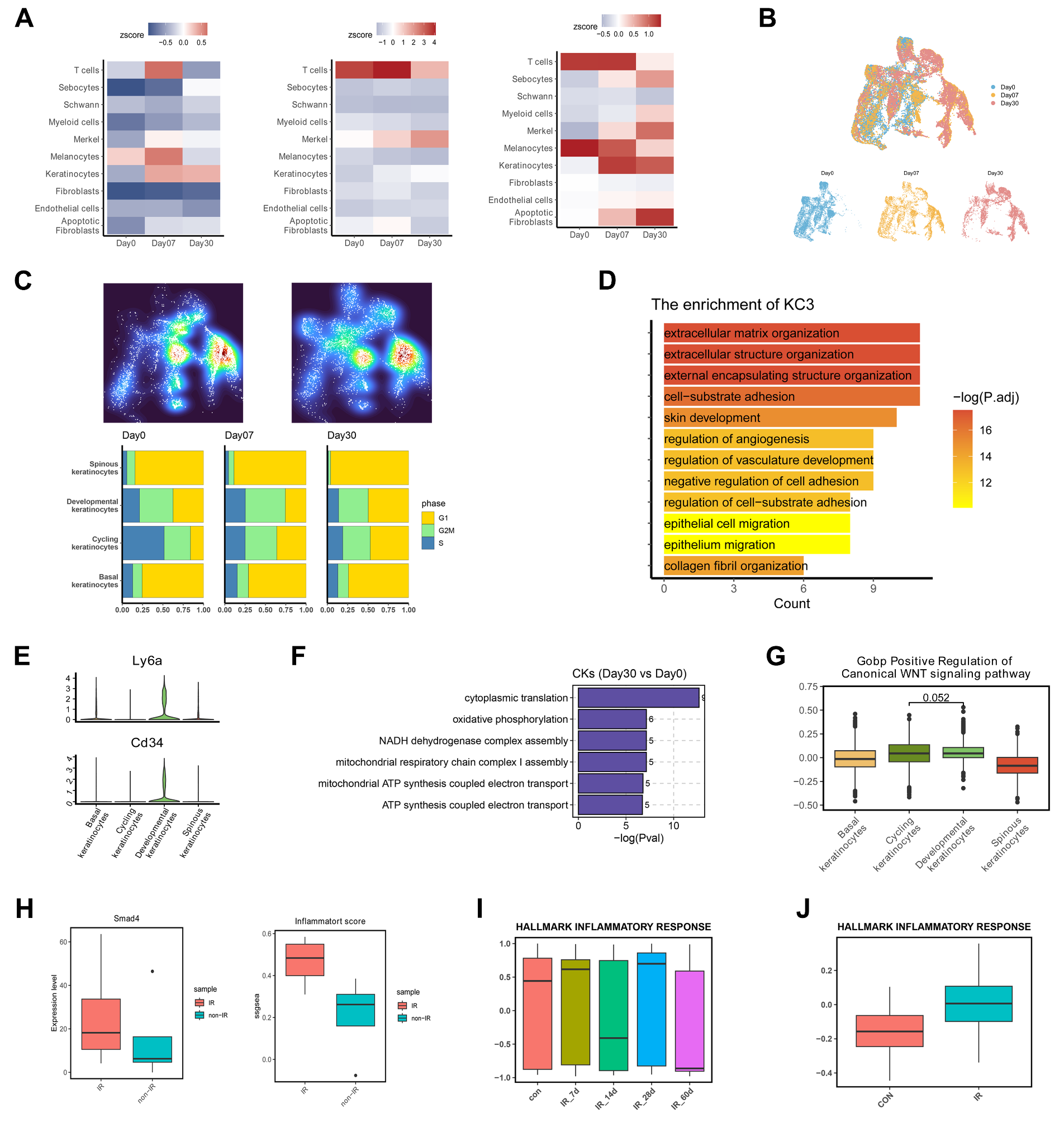
**

**Supplementary Fig. S2. Analysis of KC subsets. Corresponding to Figures 2. A.** Heat map showing the expression of Smad2, Smad3, Smad4 among 10 distinct clusters at days 0, 7, and 30 post-irradiation. The color scheme was based on z-score distribution. **B**. UMAP showing KCs composition from various groups at days 0, 7, and 30 post-irradiation. Up: merge. Down: separate. **C.** UMAP (Left: G2M state; Right: S state) and bar chart showing the relative proportions in each G1/S/G2M state of the 4 KC subclusters. **D.** Key GOBP pathways enriched by upregulated DEGs of KC3 subcluster. The redder the bar, the smaller the Padjust value. **E.** Expression of the Ly6a and Cd34 mRNA across various KC cell subtypes, visualized in violin plot. **F.** Key GOBP pathways enriched by upregulated DEGs in cycling keratinocyte across days 30, comparing days 0. The numbers on the columns represented the quantity of DEGs associated with this pathway. **G.** Box plot showing the ssGSEA indicated the role of the 4 KC subtypes in “Gobp Positive Regulation of Canonical WNT signaling pathway” on day 7 post-irradiation. **H.** The Smad4 (left) and inflammatory score calculated using "HALLMARK_INFLAMMATORY_RESPONSE" gene set (right) in 8 mouse skin samples from irradiation and non-irradiation groups, based on GSE130183 bulk RNA sequencing data. **I.** The score of cycling keratinocytes calculated using "HALLMARK_INFLAMMATORY_RESPONSE" gene set in the rat single-cell RNA sequencing dataset at Days 0, 7, 14, 28, and 60 post-irradiation (GSE193564). **J.** The score of cycling keratinocytes calculated using "HALLMARK_INFLAMMATORY_RESPONSE" gene set in the human single-cell RNA sequencing dataset from irradiation and non-irradiation groups (GSE193807). Day0: without radiotherapy. Day7: 7 days after radiotherapy. Day30: 30 days after radiotherapy.

**
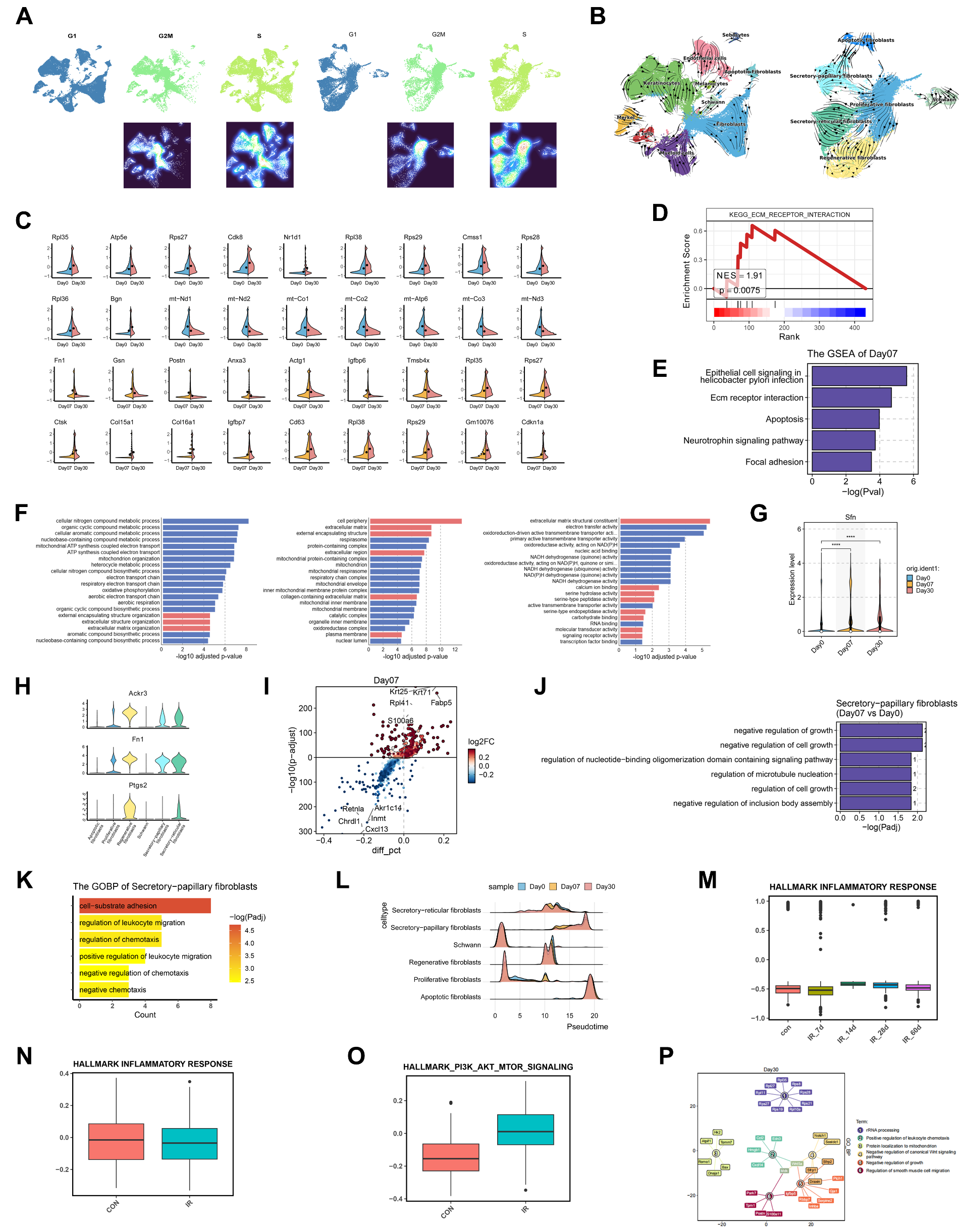
**

**Supplementary Fig. S3. Analysis of Fib subsets. Corresponding to Figures 3. A.** UMAP showing the relative proportions in each G1/S/G2M state of the total clusters (left) and the fibroblast subclusters (right). **B.** RNA velocity analysis for the total 10 clusters (up) and the 6 fibroblast subclusters (down). Arrows show the local average velocity evaluated on a regular grid, indicating the extrapolated future states of cells. **C.** Violin plot showing DEGs of Fibs in control/30 days post-RT group comparison (up) as well as 7 days post-RT/30 days post-RT group comparison (down). The black point in each image represented the mean value for the corresponding group, calculated using the summarySE function. **D.** GSEA indicated that ECM RECEPTOR INTERACTION was highly enriched in the 7 days post-RT group (red), comparing day 0 (blue). **E.** Key GOBP pathways enriched by upregulated DEGs of the 7 days post-RT group. **F.** GOAT showing the top 20 terms of GO_BP (left), GO_CC (center), and GO_MF (right) enriched by upregulated DEGs of the 7 days post-RT group, comparing day 0. The up-regulated terms were colored in red, while the down-regulated terms were colored in blue. **G.** Expression of the Sfn among various groups at days 0, 7, and 30 post-irradiation of Fib cells, visualized in violin plot. **H.** Expression of the Ackr3, Fn1, and Ptgs2 mRNA among different subtypes of Fib cells, visualized in violin plot. **I.** Volcano plot showing DEGs of secretory-papillary fibroblasts across day 7, comparing day 0. The genes shown in red indicate upregulation, while those in blue represent downregulation at day 7 post-irradiation, comparing day 0. **J.** Key GOBP pathways enriched by upregulated DEGs of secretory-papillary fibroblasts, comparing days 7 and 0. The numbers on the columns represented the quantity of DEGs associated with this pathway. **K.** Key GOBP pathways enriched by upregulated DEGs of secretory-papillary fibroblasts. The redder the bar, the smaller the Padjust value. **L.** Pseudotime analysis of the 6 Fib subsets at days 0, 7, and 30 post-irradiation. **M.** The score of secretory-papillary fibroblasts calculated using "HALLMARK_INFLAMMATORY_RESPONSE" gene set in the rat single-cell RNA sequencing dataset at Days 0, 7, 14, 28, and 60 post-irradiation (GSE193564). **N.** The score of secretory-papillary fibroblasts calculated using "HALLMARK_INFLAMMATORY_RESPONSE" gene set in the human single-cell RNA sequencing dataset from irradiation and non-irradiation groups (GSE193807). **O.** The score of secretory-papillary fibroblasts calculated using "HALLMARK_PI3K_AKT_MTOR_SIGNALING" gene set in the human single-cell RNA sequencing dataset from irradiation and non-irradiation groups (GSE193807). **P.** Network showing the top DEGs and pathways in Apoptotic Fibs across days 30. Day0: without radiotherapy. Day7: 7 days after radiotherapy. Day30: 30 days after radiotherapy.

**
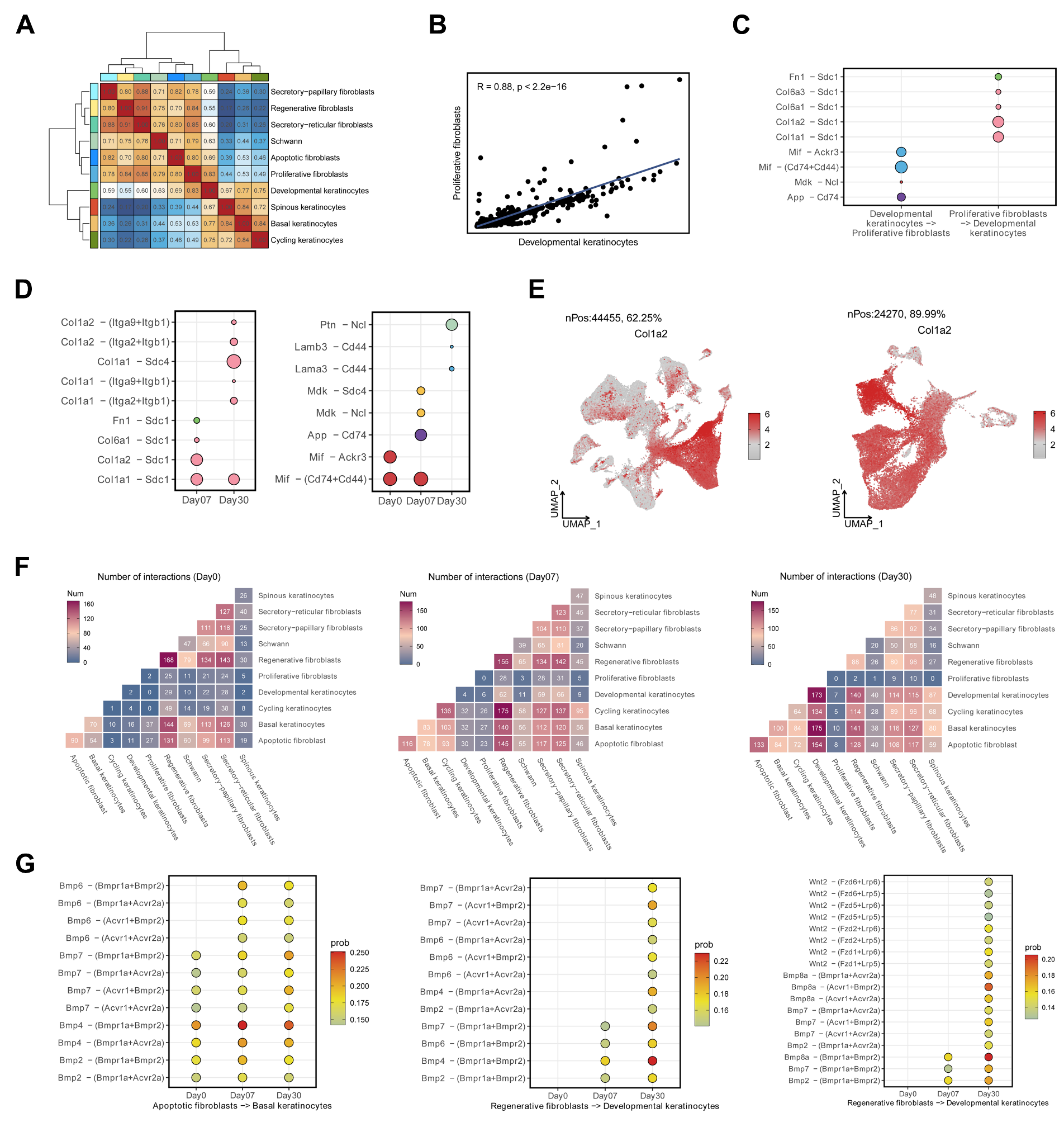
**

**Supplementary Fig. S4. Cellular crosstalk between Fibs and KCs subclusters. A.** Heatmap showing the correlation of gene expression among each KC and Fib subcluster in RISI. **B.** Pearson correlation analysis of developmental keratinocytes with proliferative fibroblasts. **C.** The interaction between developmental keratinocytes and proliferative fibroblasts from the entire experimental groups. In the context of receptor-ligand relationships between the two subpopulations, the arrow pointed from the ligand to the receptor (as shown below the figure). For receptor-ligand relationships involving genes, the ligand was typically indicated on the left side of the dash, while the receptor was positioned on the right of the dash, sometimes enclosed within parentheses (as depicted in the vertical coordinates). **D.** The interaction between developmental keratinocytes and proliferative fibroblasts in each group at days 0, 7, and 30 following irradiation (left and right). **E.** UMAP plots of Col1a2 in the total clusters (left) and the fibroblast subclusters (right). **F.** Heatmap showing the interaction intensity among each KC and Fib subcluster in RISI. **G.** The interactions of Apoptotic fibroblasts with Basal keratinocytes, Apoptotic fibroblasts with developmental keratinocytes, and Regenerative fibroblasts with Developmental keratinocytes via Wnt/Bmp interaction pairs in mouse skin samples at days 0, 7, and 30 post-irradiation. Day0: without radiotherapy. Day7: 7 days after radiotherapy. Day30: 30 days after radiotherapy.

**
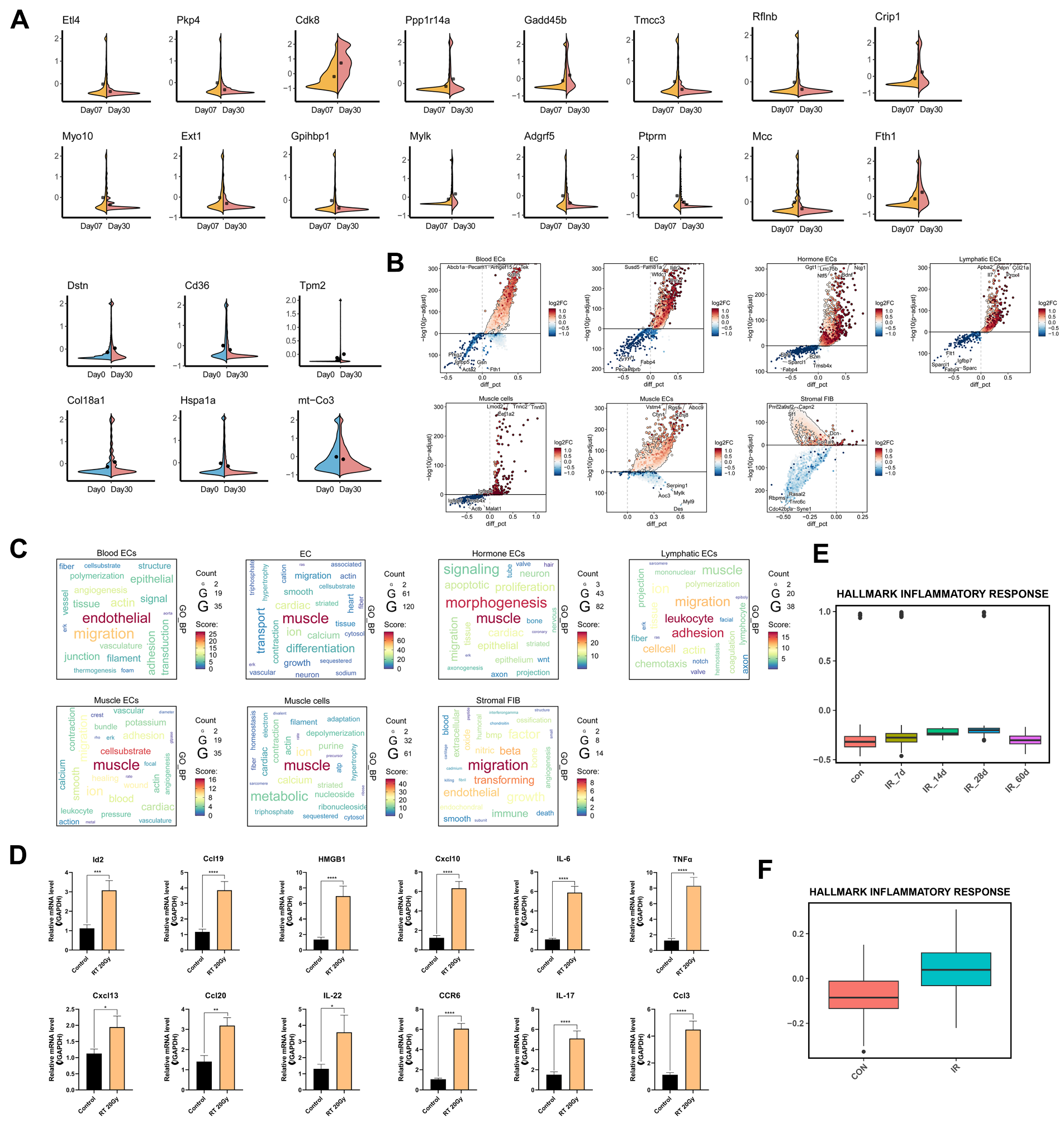
**

**Supplementary Fig. S5. Analysis of EC subsets. Corresponding to Figures 4.** **A.** Violin plot showing DEGs of ECs in 7 days post-RT/30 days post-RT group comparison (up) as well as control/30 days post-RT group comparison (down). The black point in each image represented the mean value for the corresponding group, calculated using the summarySE function. **B.** Volcano plot showing the Key DEGs enriched in each EC subcluster. The genes shown in red indicate upregulation, while those in blue represent downregulation in each EC subcluster, comparing the other EC subclusters. **C.** Wordcloud showing the Key character enriched by upregulated DEGs in each EC subcluster. **D.** The mRNA expression levels of the inflammatory signatures (Id2, Ccl19, HMGB1, Cxcl10, IL-6, TNFα, Cxcl13, Ccl20, IL-22, CCR6, IL-17, Ccl3) from skin tissues of C57BL/6 with or without radiation of 20Gy. **E.** The score of Lymphatic ECs calculated using "HALLMARK_INFLAMMATORY_RESPONSE" gene set in the rat single-cell RNA sequencing dataset at Days 0, 7, 14, 28, and 60 post-irradiation (GSE193564). **F.** The score of Lymphatic ECs calculated using "HALLMARK_INFLAMMATORY_RESPONSE" gene set in the human single-cell RNA sequencing dataset from irradiation and non-irradiation groups (GSE193807). Day0: without radiotherapy. Day7: 7 days after radiotherapy. Day30: 30 days after radiotherapy.

**
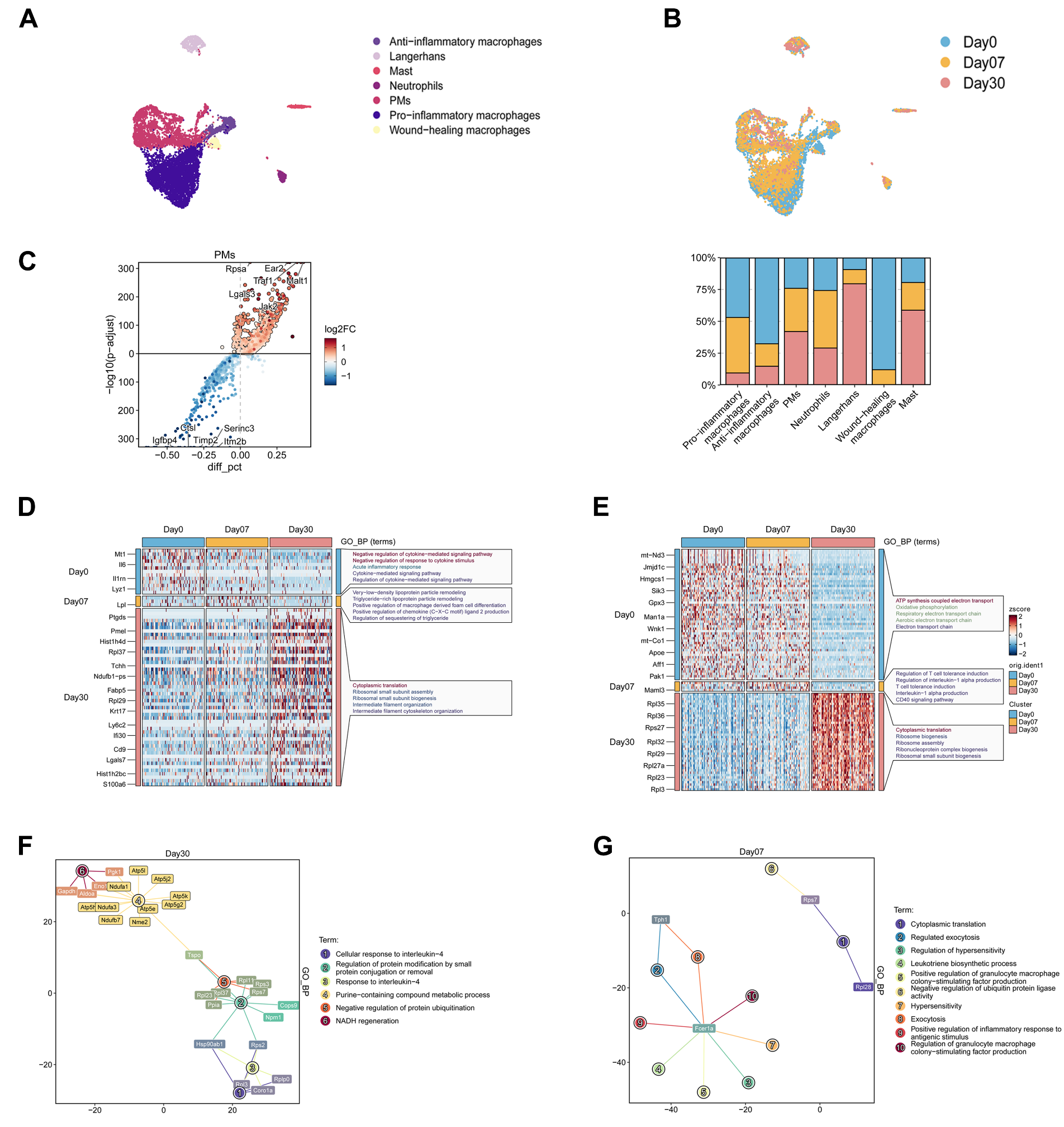
**

**Supplementary Fig. S6. Analysis of myeloid cell subsets. Corresponding to Figures 6.** **A.** UMAP visualization of 7 myeloid cell subclusters. **B.** UMAP showing myeloid cell composition from various groups at days 0, 7, and 30 post-irradiation (up); Bar plot showing the relative contribution of the three states to the total number of each myeloid cell subcluster (down). Non-repetitive sampling of 1,000 cells at each time point, including days 0, 7, and 30 post-irradiation, was conducted to eliminate the influence of cell number. **C.** Volcano plot showing the Key DEGs enriched in PMs. The genes shown in red indicate upregulation, while those in blue represent downregulation in PMs, comparing the other myeloid cell subclusters. **D.** Heat map showing the most differentially expressed genes (left) and representative GO pathway enriched by upregulated DEGs in PMs at days 0, 7, and 30 post-irradiation. **E.** Heat map showing the most differentially expressed genes (left) and Key GO pathway enriched by upregulated DEGs in Langerhans at days 0, 7, and 30 post-irradiation. **F.** Network showing the top DEGs and pathways in Th17 across days 30. **G.** Network showing the top DEGs and pathways in Mast cells across days 7. Day0: without radiotherapy. Day7: 7 days after radiotherapy. Day30: 30 days after radiotherapy.

**
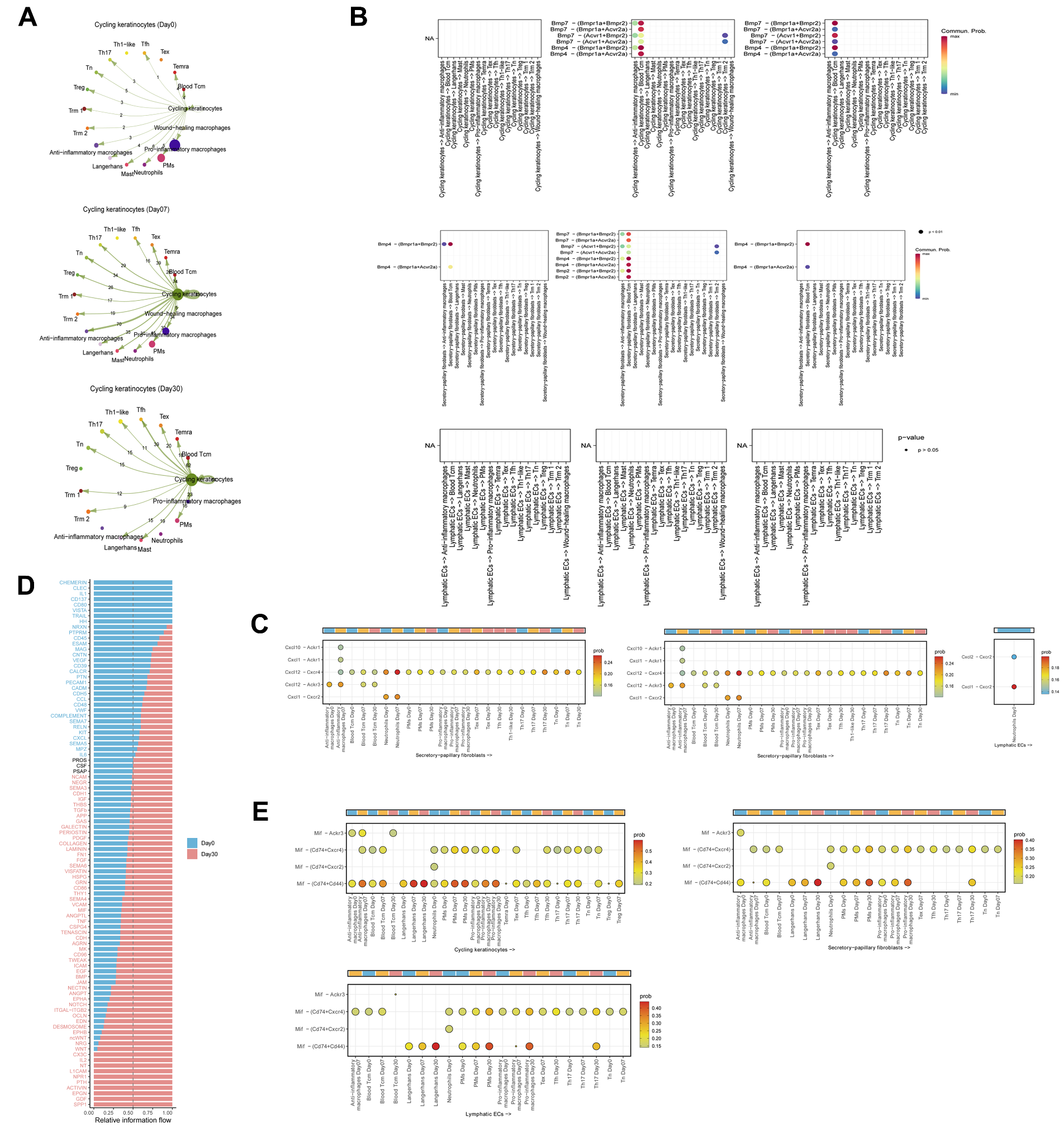
**

**Supplementary Fig. S7. Regulatory network among KCs, Fibs, and ECs subclusters with immune cells. Corresponding to Figures 7. A.** Network view of the strength of signaling between cycling keratinocytes and immune cells at days 0 (up), 7 (medium), and 30 (down) post-irradiation. The number on the lines represents the strength of signaling. In the context of receptor-ligand relationships between subpopulations, the arrow pointed from the ligand to the receptor. **B.** The interactions among cycling keratinocytes and Secretory-papillary fibroblasts with immune cells via Bmp interaction pairs in mouse skin samples at days 0, 7, and 30 post-irradiation. In the context of receptor-ligand relationships between subpopulations, the arrow pointed from the ligand to the receptor (as shown below the figure). For receptor-ligand relationships involving genes, the ligand was typically indicated on the left side of the dash, while the receptor was positioned on the right of the dash, sometimes enclosed within parentheses (as depicted in the vertical coordinates). **C.** The interaction among cycling keratinocytes, Secretory-papillary fibroblasts, and Lymphatic ECs with immune cells via Cxcl interaction pairs in mouse skin samples at days 0, 7, and 30 post-irradiation. The color above the figure represented various time points of the samples. Same meaning as the previous colors, day0 (blue), day7 (orange), and day30 (pink). **D.** The relativeinformation flow of secreting signals in the control and 30 days post-irradiation groups. **E.** The interaction of cycling keratinocytes, Secretory-papillary fibroblasts, and Lymphatic ECs with immune cells via Mif interaction pairs in mouse skin samples at days 0, 7, and 30 post-irradiation. Day0: without radiotherapy. Day7: 7 days after radiotherapy. Day30: 30 days after radiotherapy.

**Supplementary Table S1. Group and treatment information of mice.**

|  | Day0 | Day7 | Day30 |
| --- | --- | --- | --- |
| First inspection | 1 sample-3 mice  (13,823 cells) |  |  |
| Second inspection | 1 sample-3 mice  (10,704 cells) | 2 samples-6 mice  (12,395 cells;11,101 cells) |  |
| Third inspection | 1 sample-3 mice  (12,437 cells) |  | 1 sample-3 mice  (10,952 cells) |

**Supplementary Table S2. The cell cycle reference genes.**

| G1/S genes | Mcm4, Exo1, Slbp, Gmnn, Cdc45, Msh2, Mcm6, Rrm2, Pold3, Blm, Ubr7, Mcm5, Clspn, Hells, Nasp, Rpa2, Rad51ap1, Tyms, Rrm1, Rfc2, Prim1, Brip1, Usp1, Ung, Pola1, Mcm2, Fen1, Tipin, Pcna, Cdca7, Uhrf1, Casp8ap2, Cdc6, Dscc1, Wdr76, E2f8, Dtl, Ccne2, Atad2, Gins2, Chaf1b, Pcna-ps2 |
| --- | --- |
| G2M genes | Nuf2, Psrc1, Ncapd2, Ccnb2, Smc4, Lbr, Tacc3, Cenpa, Kif23, Cdca2, Anp32e, G2e3, Cdca3, Anln, Cenpe, Gas2l3, Tubb4b, Cenpf, Dlgap5, Hjurp, Cks1brt, Gtse1, Bub1, Birc5, Ube2c, Rangap1, Hmmr, Ect2, Tpx2, Ckap5, Cbx5, Nek2, Ttk, Cdca8, Nusap1, Ctcf, Cdc20, Cks2, Mki67, Tmpo, Ckap2l, Aurkb, Kif2c, Cdk1, Kif20b, Top2a, Aurka, Ckap2, Hmgb2, Cdc25c, Ndc80, Kif11 |

**Supplementary Table S3. Primers used for qPCR.**

| Gene | Forward 5'→3' | Reverse 5'→3' |
| --- | --- | --- |
| *IL-17* | GGAAAGCTGGACCACCACA | CACACCCACCAGCATCTTCTC |
| *CCL20* | TTGCTTTGGCATGGGTACTG | TCGGCCATCTGTCTTGTGAA |
| *CCL3* | TTCTCTGTACCATGACACTCTGC | CGTGGAATCTTCCGGCTGTAG |
| *CCR6* | ATGCGGTCAACTTTAACTGTGG | CCCGGAAAGATTTGGTTGCCT |
| *IL-22* | TCCGAGGAGTCAGTGCTAAA | AGAACGTCTTCCAGGGTGAA |
| *Id2* | CTCCTGGTGAAATGGCTGAT | GCTTATGTCGAATGATAGCAAAG |
| *Ccl19* | CCTGGGAACATCGTGAAAGC | TAGTGTGGTGAACACAACAGC |
| *Ccl21* | GTGATGGAGGGGGTCAGGA | GGGATGGGACAGCCTAAACT |
| *Cxcl10* | ATGACGGGCCAGTGAGAATG | TCGTGGCAATGATCTCAACAC |
| *Cxcl13* | TCTCCAGGCCACGGTATTCT | GGGGCGTAACTTGAATCCGA |
| *Tnfsf15* | GACTGTATGCTTCGGGCCAT | ATTGTCAGGTGTGCTCTCGG |
| *IFN-β* | TGGGAGATGTCCTCAACTGC | CCAGGCGTAGCTGTTGTACT |
| *HMGB1* | TTTCAAACAAAGATGCCACA | GTTCCCTAAACTCCTAAGCAGATA |
| *TNF-α* | CTTCTCATTCCTGCTTGTG | ACTTGGTGGTTTGCTACG |
| *IL-6* | TCCAGTTGCCTTCTTGGGAC | GACAGGTCTGTTGGGAGTGG |
| *GAPDH* | AGGTCGGTGTGAACGGATTTG | TGTAGACCATGTAGTTGAGGTCA |

**Supplementary Table S4. The final dataset of cells.**

| Name | Frequency | SUM |
| --- | --- | --- |
| Basal keratinocytes | 10268 |  |
| Cycling keratinocytes | 5077 |  |
| Developmental keratinocytes | 2951 |  |
| Spinous keratinocytes | 4368 | 22664 |
| Proliferative fibroblasts | 9876 |  |
| Regenerative fibroblasts | 5262 |  |
| Secretory-papillary fibroblasts | 3945 |  |
| Secretory-reticular fibroblasts | 5668 |  |
| Apoptotic fibroblasts | 1114 |  |
| Schwann | 1106 | 26971 |
| Blood ECs | 1850 |  |
| EC | 779 |  |
| Hormone ECs | 212 |  |
| Lymphatic ECs | 451 |  |
| Muscle cells | 109 |  |
| Muscle ECs | 798 |  |
| Stromal FIB | 3120 | 7319 |
| Blood Tcm | 210 |  |
| Temra | 56 |  |
| Tex | 89 |  |
| Tfh | 104 |  |
| Th1-like | 240 |  |
| Th17 | 338 |  |
| Tn | 316 |  |
| Treg | 60 |  |
| Trm 1 | 416 |  |
| Trm 2 | 200 | 2029 |
| Langerhans | 472 |  |
| PMs | 2797 |  |
| Mast | 222 |  |
| Neutrophils | 252 |  |
| Pro-inflammatory macrophages | 3785 |  |
| Anti-inflammatory macrophages | 495 |  |
| Wound-healing macrophages | 215 | 8238 |
| Sebocytes | 636 |  |
| Melanocytes | 615 |  |
| Merkel | 2940 | 4191 |
